# Combined poly-ADP ribose polymerase and ataxia-telangiectasia mutated/Rad3-related inhibition targets ataxia-telangiectasia mutated-deficient lung cancer cells

**DOI:** 10.1038/s41416-019-0565-8

**Published:** 2019-09-04

**Authors:** Nicholas R. Jette, Suraj Radhamani, Greydon Arthur, Ruiqiong Ye, Siddhartha Goutam, Anthony Bolyos, Lars F. Petersen, Pinaki Bose, D. Gwyn Bebb, Susan P. Lees-Miller

**Affiliations:** 1 Departments of Biochemistry and Molecular Biology, Robson DNA Science Centre and Charbonneau Cancer Institute, Cumming School of Medicine, University of Calgary, 3330 Hospital Drive NW, Calgary, Alberta T2N 1N4 Canada; 2 Department Oncology, Robson DNA Science Centre and Charbonneau Cancer Institute, Cumming School of Medicine, University of Calgary, 3330 Hospital Drive NW, Calgary, Alberta T2N 1N4 Canada

**Keywords:** Cancer, Non-small-cell lung cancer, Lung cancer

## Abstract

**Background:**

Up to 40% of lung adenocarcinoma have been reported to lack ataxia-telangiectasia mutated *(ATM)* protein expression. We asked whether ATM-deficient lung cancer cell lines are sensitive to poly-ADP ribose polymerase (PARP) inhibitors and determined the mechanism of action of olaparib in ATM-deficient A549 cells.

**Methods:**

We analysed drug sensitivity data for olaparib and talazoparib in lung adenocarcinoma cell lines from the Genomics of Drug Sensitivity in Cancer (GDSC) project. We deleted ATM from A549 lung adenocarcinoma cells using CRISPR/Cas9 and determined the effects of olaparib and the ATM/Rad3-related (ATR) inhibitor VE-821 on cell viability.

**Results:**

IC_50_ values for both olaparib and talazoparib positively correlated with *ATM* mRNA levels and gene amplification status in lung adenocarcinoma cell lines. ATM mutation was associated with a significant decrease in the IC_50_ for olaparib while a similar trend was observed for talazoparib. A549 cells with deletion of ATM were sensitive to ionising radiation and olaparib. Olaparib induced phosphorylation of DNA damage markers and reversible G2 arrest in ATM-deficient cells, while the combination of olaparib and VE-821 induced cell death.

**Conclusions:**

Patients with tumours characterised by ATM-deficiency may benefit from treatment with a PARP inhibitor in combination with an ATR inhibitor.

## Background

A key goal of precision oncology is to target cancer cells based on characteristics that are unique to that tumour, thus minimising deleterious effects on normal tissue. One recent and striking example of the success of this approach is targeting DNA repair-deficient cancers with poly-ADP ribose polymerase (PARP) inhibitors. Cells with defects in genes required for repair of DNA double-strand breaks (DSBs) via the homologous recombination repair (HRR) pathway are exquisitely sensitive to PARP inhibitors,^1,2^ and the PARP inhibitors olaparib, rucaparib and niraparib have been approved for use in patients with BRCA-deficient breast and/or ovarian cancers, while talazoparib and veliparib are in clinical trials.^3–6^

Importantly, synthetic lethality with PARP inhibitors may not be limited to cells with mutation in the *BRCA* genes, as cells with depletion of other DNA damage response proteins, including ataxia-telangiectasia mutated (ATM) are also sensitive to PARP inhibition.^3,7,8^ ATM is a member of the phosphatidylinositol-3 kinase-like (PIKK) family of serine/threonine protein kinases and plays a critical role in regulating the cellular response to DNA damage.^9–11^ Activation of ATM leads to phosphorylation of many downstream targets that together regulate DSB repair pathway choice, cell cycle checkpoints, DSB repair in heterochromatin and other cellular processes.^9,12–14^ Loss of both copies of the *ATM* gene leads to ataxia-telangiectasia, a devastating childhood condition characterised by cerebellar degeneration, progressive loss of neuromuscular control, cancer predisposition, immune defects and telangiectasia.^15^ Additionally, many human cancers harbour somatic mutations in *ATM*, including ~40% of mantle cell lymphoma (MCL),^16,17^ ~18% of colorectal cancers^18^ and over 10% of lung^19^ and prostate^20^ cancers, raising the possibility that ATM-deficient tumours can be targeted with radiation, chemotherapy and/or novel therapies such as PARP inhibitors.^21^ Indeed, we previously showed that ATM-deficient MCL,^22,23^ colorectal^24^ and gastric cancer^25^ cell lines with depletion or inhibition of ATM are sensitive to olaparib, especially when accompanied by loss or mutation of *TP53.*^22,24,25^ We also demonstrated that relative loss of ATM has clinical implications, conferring worse outcome and associated with improved benefit from cisplatin therapy.^26^

Another potential target for PARP inhibitor therapy is lung cancer. Lung cancer is the leading cause of cancer death worldwide. Approximately 40% of lung cancers are adenocarcinoma, while 30% are squamous cell carcinoma and 15% small cell carcinoma. The frequency of somatic mutations in the *ATM* gene in lung adenocarcinoma is estimated to be ~11%.^27,28^ Approximately 57% of *ATM* mutations are mis-sense, while 41% are predicted to result in truncation of the ATM protein.^27,28^ Of note, it has been reported that over 40% of lung adenocarcinoma are negative for ATM protein staining by immunohistochemistry.^29^ Moreover, deletion of *ATM* improved radiation response^30^ and sensitivity to PARP inhibitors in mouse models of lung cancer,^31^ making ATM-deficient lung cancer a potential target for both traditional and novel therapeutics, such as PARP inhibitors.

Optimal use of PARP inhibitors as therapeutic agents requires a thorough understanding of their mechanism of action and the effects of modifying factors on PARP inhibitor susceptibility. PARP proteins are involved in a wide range of cellular processes.^32,33^ The most well-studied member of the PARP family, PARP-1, mediates DSB repair through alternative non-homologous end joining (a-NHEJ) and facilitates repair of single-stranded DNA (ssDNA) breaks.^34,35^ PARP also assists in repair of ssDNA breaks at replication forks through poly-ADP-ribosylation (PARylation) of target proteins.^35^ PARP inhibitors were originally proposed to act by inhibiting base excision repair, thus enhancing production of DSBs when cells attempted DNA replication. However, later studies questioned this role, and subsequently PARP inhibitors such as olaparib were shown to induce replication fork collapse, accumulation of DNA damage and cell death.^8,36,37^ PARP inhibitors have also been shown to cause uncontrolled acceleration of replication fork threshold speed, giving cells less time for DNA repair leading to accumulation of ssDNA breaks and reduction in cell survival.^38^ Recently inhibition of poly-ADP ribose glycohydrolase (PARG), the enzyme that removes poly-ADP ribose (PAR), was shown to induce PARylation at unligated Okazaki fragments, further supporting a role for PARP in DNA replication.^39^ Mechanistically, olaparib induces DNA damage (as revealed by histone H2AX phosphorylation,^40,41^) G2 arrest,^42^ decreased proliferation^38^ and cell death^42^ in a variety of cell types.

How PARP inhibitors selectively target ATM-deficient cells is poorly understood. In ATM-deficient cells, olaparib has been shown to induce replication-dependent phosphorylation of histone H2AX,^40,42,43^ autophosphorylation of DNA-dependent protein kinase catalytic subunit (DNA-PKcs) on serine 2056,^44,45^ phosphorylation of p53 on serine 15 and upregulation of p21.^22^ In bladder cancer cells, olaparib was shown to induce reactive oxygen species (ROS) and ROS production was potentiated in the absence of ATM,^43^ suggesting that olaparib can induce ROS-mediated cell death.

To better understand the potential for targeting ATM-deficient lung cancer with PARP inhibitors, we studied the association between PARP inhibitor sensitivity and *ATM* status in 61 lung adenocarcinoma cell lines from the Genomics of Drug Sensitivity in Cancer (GDSC) project. We found that mis-sense mutations in *ATM* and low *ATM* gene expression were associated with increased sensitivity to olaparib, while low ATM expression correlated with sensitivity to talazoparib. Conversely, *ATM* gene amplification was associated with reduced sensitivity to both olaparib and talazoparib. Based on these data, we deleted either ATM or the related protein kinase DNA-PKcs/*PRKDC* from A549 lung adenocarcinoma cell lines using CRISPR/Cas9, and used these cells to examine the mechanism of action of olaparib. As expected, ATM-deficient A549 cells were sensitive to both ionising radiation (IR) and olaparib. Mechanistically, olaparib induced autophosphorylation of ATM and DNA-PKcs, as well as DNA-PK-dependent phosphorylation of γ-H2AX and p53. Surprisingly, in ATM-deficient A549 cells, olaparib induced transient, reversible G2 checkpoint arrest but not cell death. However, the addition of VE-821, an inhibitor of the related PIKK, ATM and Rad3-related (ATR),^46^ significantly enhanced cell death in olaparib-treated ATM-deficient cells with little effect on ATM-proficient cells. Our study sheds light on the mechanism of action of PARP inhibitors in ATM-deficient cells and reveals that the addition of an ATR inhibitor is required to induce cell death in olaparib-treated ATM-deficient lung cancer cells. Together, these results suggest that the combination of a PARP inhibitor with an ATR inhibitor may have significance for the treatment of patients with ATM-deficient cancers.

## Methods

### Analysis of publicly available data on olaparib and talazoparib sensitivity in lung cancer

Processed IC_50_ and area under the curve (AUC) drug sensitivity data, robust multi-array average (RMA) normalised gene expression data, processed whole-exome sequencing (WES) variant calls, copy number data derived from PICNIC,^47^ Affymetrix SNP6 array segmentation files, and cell line annotation data were downloaded from the GDSC website (https://www.cancerrxgene.org/downloads). Analyses were restricted to cell lines corresponding to the Cancer Genome Atlas (TCGA) code “LUAD” (Supplementary Table 1). ENSEMBL gene identifiers were converted to Human Genome Organization (HUGO) gene symbols using the biomaRt Bioconductor package.^48^ Pearson correlations were performed between ln(IC_50_) values of olaparib/talazoparib and *ATM* gene expression values. The correlation between *ATM* gene expression and olaparib/talazoparib IC_50_ values was visualised using scatterplots. Associations with *ATM* mutation and gene amplification were visualised using box plots. Cell lines containing both amplifications and mutations of the *ATM* gene were excluded from the analysis. Boxplots and scatterplots were plotted using the ggplot2 package.^49^ Bioinformatics analyses were performed using the R programming language.

### Cell lines

A549 parental cells were purchased from the American Type Culture Collection (ATCC) and were cultured in Dulbecco’s Modified Eagle Medium (DMEM) (ThermoFisher Scientific, MA, USA) in the presence of 50 µg/mL penicillin–streptomycin (Gibco, ThermoFisher Scientific) and 10% (w/v) Hyclone Fetalclone III Serum in 100 mm sterile plastic dishes. Cells were grown in a humidified incubator under 5% CO_2_ at 37 °C. A549 cells with CRISPR/Cas9-induced loss of expression of ATM, DNA-PKcs or control cells were generated as described in Supplementary Information.

### Inhibitors

Olaparib, the DNA-PK inhibitor NU7441 and VE-821 were purchased from Selleck Chemicals, Texas, USA, catalogue numbers 21060, S2638 and S8007, respectively.

### Additional methods

Methods for generation of CRISPR cell lines, western blot, clonogenic survival, trypan blue, cell cycle, annexin, ROS and γ-H2AX foci assays are provided in Supplementary Material.

## Results

**Fig. 1:**
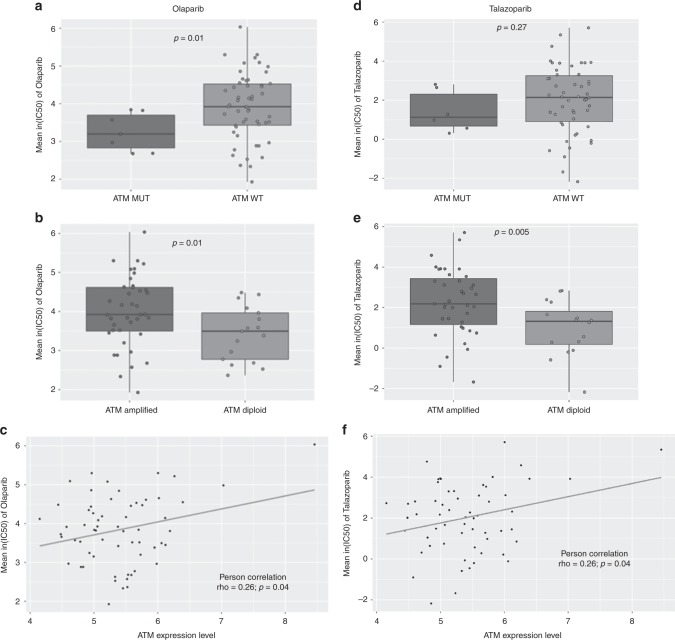
ATM deficiency is associated with PARP inhibitor sensitivity in lung adenocarcinoma cell lines described in the GDSC project. **a**, **b** Boxplots comparing olaparib IC_50_ values between (**a**) *ATM* mutant and *ATM* wild-type cells and (**b**) *ATM* amplified and *ATM* diploid cells. Cell lines containing >3 copies of the *ATM* gene were classified as “ATM amplified”. **c** Scatterplot showing the correlation between *ATM* gene expression (mRNA) and olaparib IC_50_ values. **d**–**f** ATM deficiency is associated with talazoparib sensitivity in 59 lung adenocarcinoma cell lines in the GDSC project. Boxplots comparing talazoparib IC_50_ values between (**d**) *ATM* mutant and *ATM* wild-type cells; **e** *ATM* amplified and *ATM* diploid cells. **f** Scatterplot showing the correlation between *ATM* gene expression and talazoparib IC_50_ values

Analysis of drug sensitivity data from 61 lung adenocarcinoma cell lines in the GDSC database revealed that cell lines with *ATM* mutation were significantly more sensitive to olaparib than those with wild-type *ATM* (Fig. 1a). Further, cell lines containing three or more copies of the *ATM* gene (indicated by gene amplification) were significantly less sensitive to olaparib than were cell lines that were diploid for the *ATM* gene (Fig. 1b) and *ATM* mRNA expression was higher in cell lines with amplified *ATM* (Supplementary Fig. 1). We also observed a strong positive correlation between IC_50_ values for olaparib and *ATM* gene expression (Fig. 1c and Supplementary Fig. 2). Similarly, *ATM* gene amplification and mRNA expression were inversely correlated with talazoparib sensitivity (Fig. 1e, f), and ATM mutation status trended towards talazoparib sensitivity (Fig. 1d).

**Fig. 2:**
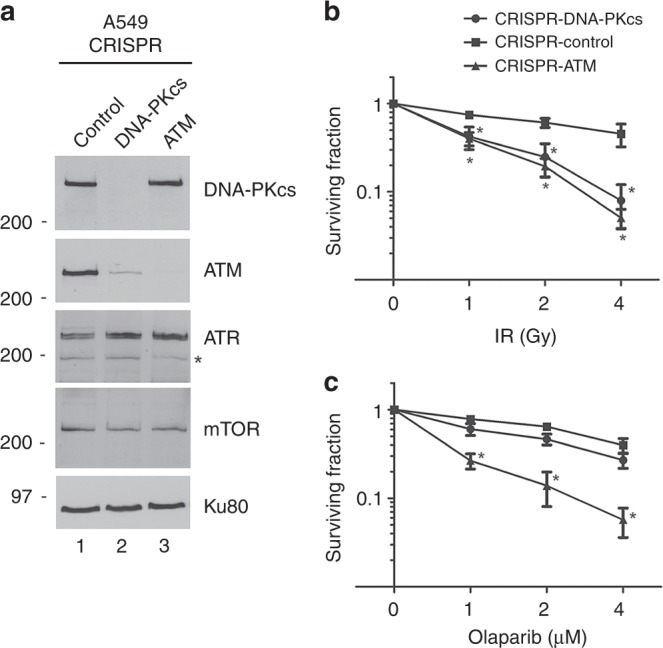
Characterisation of A549 cells with CRISPR/Cas9 depletion of ATM or DNA-PKcs. **a** Total cell extracts were prepared from A549-CRISPR-control, A549-CRISPR-DNA-PKcs cells and A549-CRISPR-ATM cells by NETN lysis. Fifty µg total protein was run on SDS-PAGE and membranes were immunoblotted for DNA-PKcs, ATM, ATR, mTOR and Ku80 (loading control) as shown on the right-hand side. The asterisk indicates a non-specific band. Positions of molecular weight markers in kDa are shown on the left-hand side. A list of antibodies used in the study are provided in Supplementary Table 2. Bands were quantitated and normalised to Ku80 as described in Supplementary Methods. The level of ATM expression in A549-CRISPR-DNA-PKcs cells was 17% of that in A549-CRISPR-control cells while the level of DNA-PKcs in A549-CRISPR-ATM cells was 93% that in control cells. Results are from 2 separate experiments. **b**, **c** A549-CRISPR-control, CRISPR-ATM and CRISPR-DNA-PKcs cell lines were treated with increasing doses of IR (**b**), or increasing concentrations of olaparib (**c**), and analysed by clonogenic survival assays as described in Methods. Results show the average of three separate experiments with each treatment carried out in triplicate. Statistical significance was determined using one-way ANOVA. Error bars represent SEM, and * indicates a *p*-value < 0.05 when compared to the control A549 cell line at the indicated time points

We previously showed that MCL,^22,23^ gastric^25^ and colorectal cancer^24^ cell lines with depletion of ATM protein were more sensitive to the PARP inhibitor olaparib than were ATM-proficient control cells, and that sensitivity to olaparib was enhanced when p53 was also deleted.^22^ Since less than 1% of lung cancer patients have mutation in both *ATM* and *TP53* (Supplementary Fig. 3), we used CRISPR/Cas9-directed mutagenesis to delete ATM and DNA-PKcs from A549 lung adenocarcinoma cells which have wild type *TP53*. Control cells were treated with vector in the absence of guide RNA (see Supplementary Methods for details). Expression of ATM and DNA-PKcs protein was determined by western blot. As expected, no ATM protein was detected in A549-CRISPR-ATM cells, while levels of DNA-PKcs were unaffected by the absence of ATM (Fig. 2a). As reported previously in tumour cells lacking DNA-PKcs, and in rodent and human cells treated with siRNA to DNA-PKcs,^50–52^ loss of DNA-PKcs resulted in an ~80% reduction in ATM protein expression (Fig. 2a). In contrast, expression of other PIKK family members, ATR and mammalian target of rapamycin (mTOR) were unaffected by DNA-PKcs loss (Fig. 2a), suggesting that reduced ATM expression is specific to loss of DNA-PKcs.

We next tested the sensitivity of the CRISPR cell lines to IR and olaparib using clonogenic survival assays. As expected, both A549-CRISPR-ATM and A549-CRISPR-DNA-PKcs cells were sensitive to IR (Fig. 2b), and A549-CRISPR-ATM cells were sensitive to olaparib (Fig. 2c). In contrast, DNA-PKcs-depleted cells, despite having low levels of ATM (Fig. 2a) were relatively resistant to olaparib (Fig. 2c), suggesting that residual ATM in these cells is sufficient to confer olaparib resistance.

During the course of these experiments, we observed that the colony size of olaparib-treated A549-CRISPR-ATM cells was consistently smaller than that of control cells. To further examine this observation, we measured the median radius of colonies in A549-CRISPR-control, A549-CRISPR-ATM and A549-CRISPR-DNA-PKcs cells treated with DMSO (control) or olaparib at 2 or 4 µM. The median colony density in all cells was reduced by incubation with olaparib, with the sizes of colonies in olaparib-treated ATM and DNA-PKcs-depleted cells being the smallest (Supplementary Fig. 4). Together, these experiments are consistent with reports that olaparib inhibits cell proliferation.^38^

To further examine the effects of olaparib on cell proliferation, control, CRISPR-DNA-PKcs and CRISPR-ATM A549 cells were incubated with either DMSO or olaparib and the number of viable cells was determined by the trypan blue exclusion assay. Addition of olaparib reduced the number of viable ATM-deficient cells at 72 h, and both control and ATM-deficient cells at 120 h (Supplementary Fig. 5). DNA-PKcs-deficient cells grew slower than either control or CRISPR-ATM A549 cells, but the apparent decrease in cell growth after olaparib treatment was not statistically significant (Supplementary Fig. 5). Additional characterisation of the A549-CRISPR-DNA-PKcs cells will be described separately (Lees-Miller et al., in preparation). Together, these results confirm that olaparib reduces cell proliferation in ATM-deficient cells.

**Fig. 3:**
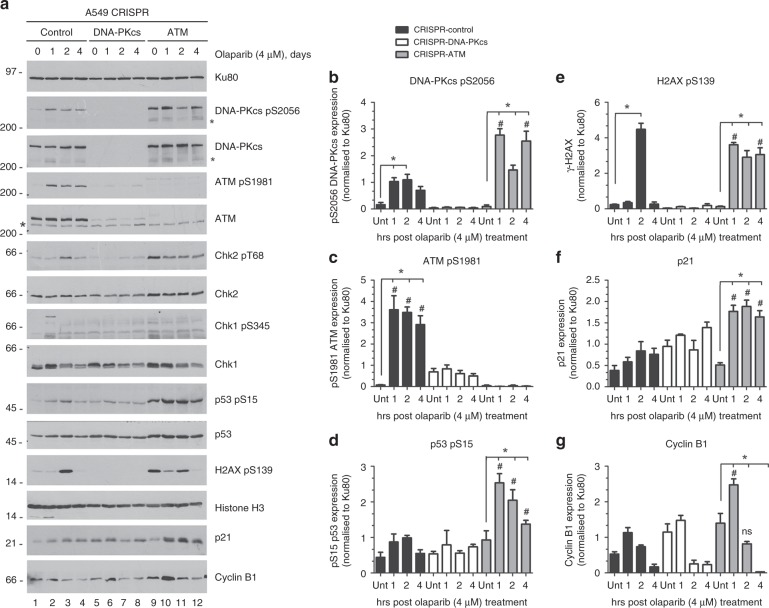
Olaparib induces a DNA damage response in ATM-deficient cells. **a** A549-CRISPR-control, A549-CRISPR-DNA-PKcs and A549-CRISPR-ATM cells were treated with olaparib (4 µM) for 1, 2 or 4 days, following which NETN extracts were generated and samples were run on SDS-PAGE as in Fig. 2a. Blots were probed for Ku80 as a loading control. Samples in lanes marked 0, were treated with DMSO for 4 days. Positions of molecular weight markers in kDa are shown on the left-hand side. The asterisks indicate non-specific bands. Panels **b**–**g** show quantitation of results from three separate experiments. Black bars represent control cells, white bars indicate A549-CRISPR-DNA-PKcs cells and grey bars represent A549-CRISPR-ATM cells. Western blots from two additional experiments, as well as quantitation of Chk2 T68 phosphorylation, is shown in Supplementary Fig. 6. Error bars represent mean with SEM of three independent experiments. One-way ANOVA was used to determine the statistical significance of three separate experiments. * Indicates *p*-value < 0.05 when compared to its own control group. # Indicates statistical significance when compared to the corresponding treatment groups of CRISPR-control cells

To determine the mechanism by which olaparib reduces cell proliferation, olaparib-treated cells were analysed for markers of the DNA damage response. A549-CRISPR-control, A549-CRISPR-ATM and A549-CRISPR-DNA-PKcs cells were either treated with DMSO (4 days) or 4 µM olaparib for 1, 2 or 4 days. Cells were harvested, whole-cell extracts generated by NETN lysis and 50 µg protein was run on SDS-PAGE and analysed by immunoblot (Fig. 3a and Supplementary Fig. 6). In control cells, olaparib induced phosphorylation of DNA-PKcs on serine 2056, an autophosphorylation site commonly used to infer DNA-PKcs activation^44,45^ as well as autophosphorylation of ATM on serine 1981^53^ (Fig. 3a, lanes 1–4 and panels b/c), consistent with olaparib inducing DNA damage. As expected, DNA-PKcs serine 2056 phosphorylation was absent in CRISPR-DNA-PKcs cells (Fig. 3a, lanes 5–8 and panel b) but unexpectedly, was enhanced in ATM-deficient cells (Panel 3a, lanes 9–12 and panel b). Olaparib-induced ATM 1981 phosphorylation was reduced in DNA-PKcs-deficient cells (Fig. 3a, lanes 5–8 and panel c), consistent with less ATM expression in DNA-PKcs deficient cells, and, as expected, was absent in CRISPR-ATM cells.

We also probed extracts for other markers of the DNA damage response, including phosphorylation of p53 on serine 15, Chk2 on threonine 68, Chk1 on serines 317 and 345 and histone H2AX on serine 139 (γ-H2AX),^54,55^ as well as for upregulation of cyclin-dependent kinase (CDK) inhibitor p21/Cip1 and CDK1 regulatory subunit cyclin B1.^54^ Olaparib-treated ATM-deficient cells showed increased phosphorylation of p53 serine 15 and γ-H2AX (Fig. 3d, e) as well as upregulation of p21 and cyclin B1 (Fig. 3a, lanes 9–12, and Fig. 3f, g). However, no significant changes in Chk2 threonine 68 phosphorylation (Supplementary Fig.  6C) or Chk1 317 and 345 phosphorylation were observed (data not shown). Interestingly, although olaparib-induced γ-H2AX phosphorylation was observed in control and ATM-deficient cells (Fig. 3a, lanes 1–4 and 9–12 and Fig. 3e), it was undetectable in CRISPR-DNA-PKcs cells suggesting that olaparib-induced γ-H2AX phosphorylation requires DNA-PKcs (Fig. 3a, e). Moreover, γ-H2AX phosphorylation in ATM-deficient cells was significantly higher than in control cells (Fig. 3a, e), raising the possibility that DNA-PKcs or ATR might be responsible for the enhanced H2AX phosphorylation in ATM-CRISPR cells.

While both ATM and DNA-PKcs contribute to H2AX phosphorylation in response to DNA damage,^56^ DNA-PKcs has been shown to carry out H2AX phosphorylation in apoptotic cells.^57,58^ Indeed, TRAIL and staurosporine-induced apoptosis generates a characteristic DNA-PK-dependent ring of γ-H2AX around the nuclear periphery that precedes apoptotic pan-nuclear H2AX staining.^57,58^ In addition, a recent study has linked DNA-PKcs to replication stress-induced, pan-nuclear H2AX phosphorylation in S-phase cells.^59^

To better understand the mechanism behind the enhanced, olaparib-induced H2AX phosphorylation in ATM-deficient cells, A549-CRISPR-control, A549-CRISPR-DNA-PKcs and A549-CRISPR-ATM cells were treated with 4 µM olaparib for 1–4 days as in Fig. 3, and analysed for γ-H2AX foci using immunofluorescence. Olaparib induced γ-H2AX foci in control cells, while no foci were detected in CRISPR-DNA-PKcs cells and a large increase in both foci number and foci intensity was observed in CRISPR-ATM cells (Supplementary Fig. 7). In contrast, no pan-nuclear or apoptotic ring staining was observed. Thus, the increased H2AX phosphorylation in ATM-deficient cells appears to be due to increased numbers of DNA damage foci rather than an increase in apoptosis or replication stress.

**Fig. 4:**
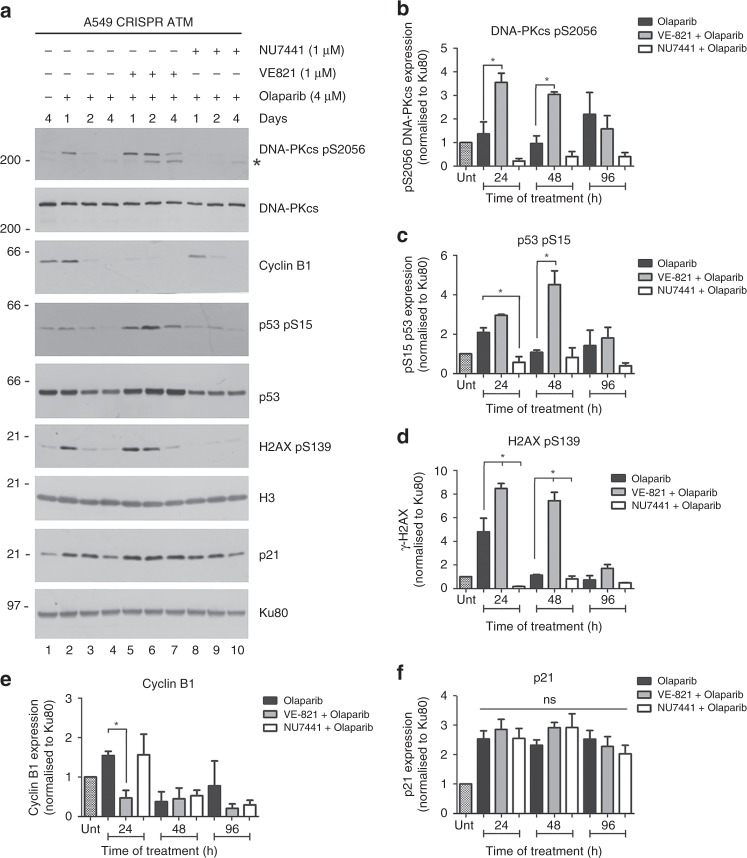
H2AX and p53 phosphorylation in olaparib-treated A549-CRISPR-ATM cells is DNA-PK-dependent. **a** A549-CRISPR-ATM cells were incubated with DMSO alone (lane 1) or olaparib (4 µM, lanes 2–10) in the absence or presence of the ATR inhibitor (VE-821, 1 µM, lanes 5–7) or the DNA-PK inhibitor (NU7441, 1 µM, lanes 8–10) as shown in lanes 5–7 and 8–10, respectively. Cells were harvested after 1, 2 or 4 days as indicated, and aliquots were run on SDS-PAGE with immunoblot as in Fig. 3. Quantitation of three separate repeats is shown in (**b**–**f**). See also Supplementary Fig. 8 for additional experimental repeats. Untreated cells are shown by the hatched bar in the first lane on the left (see Supplementary Fig. 8 for additional details). All panels show the mean with SEM from three independent experiments. One-way ANOVA was used to determine the statistical significance of three separate experiments. * indicates *p*-value < 0.05 for VE-821 + olaparib or NU7441 + olaparib treatment groups when compared with olaparib alone

To determine whether ATR contributed to phosphorylation of H2AX in olaparib-treated ATM-deficient cells, cells were incubated with olaparib (4 µM) for 1, 2 or 4 days in the presence of either the ATR inhibitor VE-821,^46^ the DNA-PK inhibitor NU7441^60^ or DMSO control, and extracts were probed for H2AX phosphorylation as above. As expected, NU4771 blocked olaparib-induced DNA-PKcs 2056 phosphorylation confirming that olaparib induces DNA-PKcs autophosphorylation (Fig. 4a, b). Moreover, olaparib-induced phosphorylation of H2AX was ablated by NU7441 but was unaffected by VE-821, confirming that DNA-PKcs is required for olaparib-induced H2AX phosphorylation in ATM-deficient cells (Fig. 4 and Supplementary Fig. 8). Similarly, olaparib-induced phosphorylation of p53 on serine 15 was also largely DNA-PKcs-dependent, whereas cyclin B1 upregulation was reduced by ATR inhibition at 24 h olaparib incubation (Fig. 4a, c and e). Together these experiments show that olaparib induces an enhanced DNA-damage response in ATM-deficient cells (compared to control cells), marked by DNA-PK-dependent autophosphorylation (serine 2056), phosphorylation of p53 (serine 15), and H2AX (serine 139), as well as upregulation of p21 and cyclin B1, pointing to activation of a cell cycle checkpoint.

**Fig. 5:**
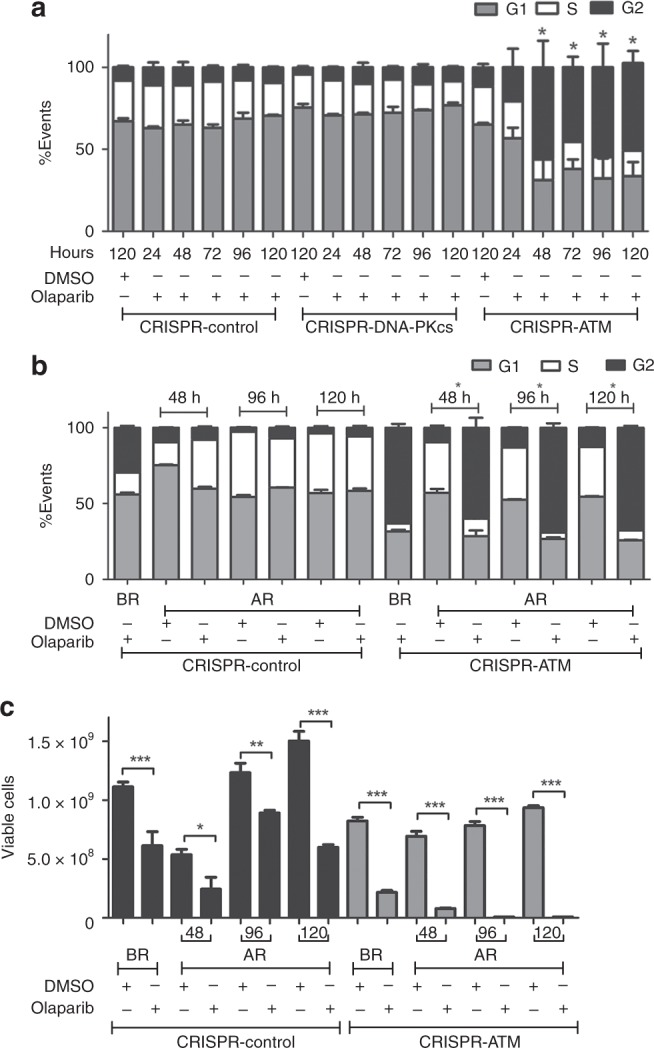
Olaparib induces reversible G2 arrest in ATM-depleted A549 cells. **a** A549-CRISPR-control, A549-CRISPR-DNA-PKcs and A549-CRISPR-ATM cells were treated with DMSO (120 h) or 4 µM olaparib and harvested after 24, 48, 72, 96 or 120 h as indicated then analysed by flow cytometry with propidium iodide staining. G1 cells are represented by grey bars, S phase cells by white bars and G2 cells by black bars. The figure shows the mean with SEM from three separate experiments. Statistical significance was determined using one-way ANOVA. * Represents, *p* < 0.05 for olaparib-treated cells relative to DMSO-treated cells. In no experiment did the sub-G1 fraction exceed 1% of total cells, indicating lack of apoptosis (data not shown). **b** A549-control and A549-CRISPR-ATM cells were treated with 4 µM olaparib for 120 h then released into fresh media containing either DMSO or 4 µM olaparib and harvested after an additional 48, 96 or 120 h, as indicated. Cells were analysed by flow cytometry with propidium iodide staining as above. The percentage of cells in G1, S and G2 are indicated in the grey, white and dark bars, respectively, as in panel (**a)**. BR indicates before release and AR, after release. The figure represents the mean with SEM of three separate experiments. Statistical significance was determined using one-way ANOVA. * Represents, *p* < 0.05 for olaparib-treated cells relative to DMSO-treated cells. **c** Cells were treated with olaparib for 120 h then released either into fresh media containing either DMSO or olaparib as indicated then analysed by trypan blue exclusion assay to determine the number of viable cells. Black bars represent A549-CRISPR-control cells and grey bars A549-CRISPR-ATM cells. The figure represents the mean with SEM of three separate experiments. Statistical significance was determined using one-way ANOVA. The * represents, *p* < 0.05 for olaparib-treated cells relative to DMSO-treated cells as above

To test for the effects of olaparib on cell cycle phase, asynchronously growing A549-CRISPR-control, A549-CRISPR-DNA-PKcs and A549-CRISPR-ATM cells were treated with olaparib (4 µM) for 24, 48, 72, 96 or 120 h then stained with propidium iodide and analysed by flow cytometry for the percentage of cells in each phase of the cell cycle (Fig. 5a). Olaparib had no significant effect on the cell cycle profile of either control or DNA-PKcs-deficient cells but induced marked G2 accumulation in ATM-deficient cells (Fig. 5a, black bars). No histone H3-S10 phosphorylation was observed in olaparib-treated ATM-deficient cells, suggesting that arrest occurred in G2 not in early mitosis (data not shown). Interestingly, in no sample did the percentage of apoptotic cells (i.e. sub-G1 DNA population) reach more than 1% of the total cells (data not shown), suggesting that olaparib does not induce cell death in ATM-deficient cells. Absence of cell death was confirmed when cells were stained for annexin as a marker of apoptosis (Supplementary Fig. 9). Thus, olaparib appears to be cytostatic rather than cytotoxic in ATM-deficient A549 cells under these conditions.

To determine whether the olaparib-induced G2 arrest was transient or sustained, cells were grown in the presence of olaparib for 120 h then released into either fresh media or fresh media containing 4 µM olaparib and analysed for cell cycle arrest by flow cytometry with propidium iodide staining (Fig. 5b) and viability using the trypan blue exclusion assay (Fig. 5c). Both G2 arrest and decreased proliferation were found to be transient in olaparib-treated ATM-deficient cells, since cells continued through the cell cycle and the number of viable cells increased when olaparib was removed (Fig. 5b, c).

ATR is essential for DNA damage-induced G2 arrest where it activates Chk1 to inhibit CDC25, preventing CDK1-cyclin A/B activation, and upregulates p53 to induce p21 expression.^61^ Given that olaparib induced transient G2 arrest in ATM-deficient A549 cells, we reasoned that addition of a G2/M checkpoint inhibitor would abrogate the G2 checkpoint and induce cell death. Indeed, the ATR inhibitors NU6027 and VE-821 have been shown to increase the cytotoxic effects of the PARP inhibitors rucaparib and veliparib in various cancer cell lines,^62–64^ and resistance of Schlafen 11-deficient cells to PARP inhibitors is overcome by inhibition of ATR^65^ but, to our knowledge, the effects of ATR inhibition on olaparib-treated ATM-deficient cells has not been determined.

**Fig. 6:**
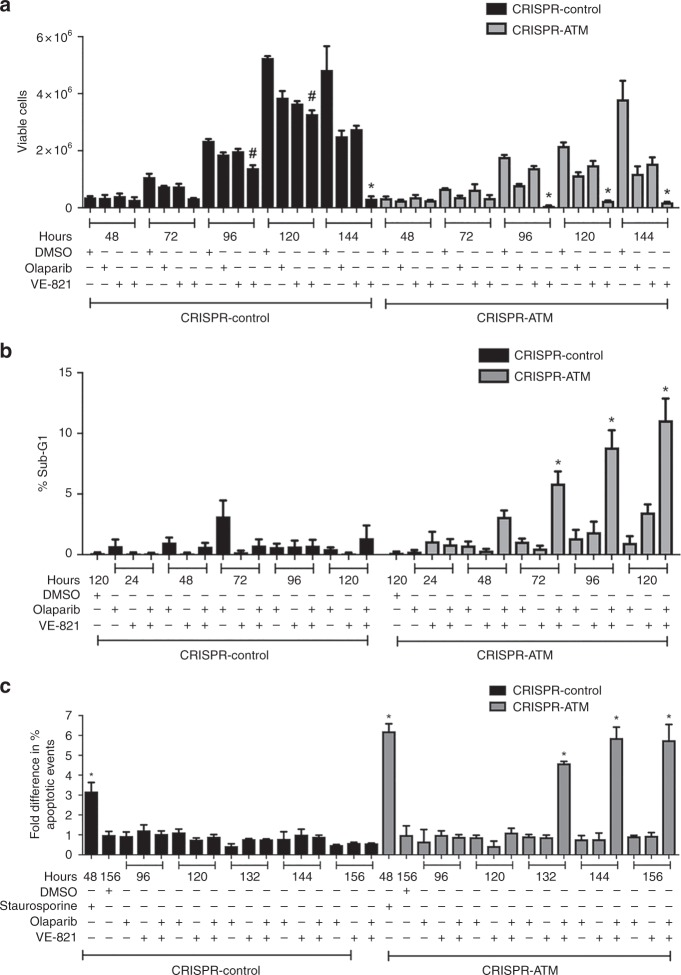
The ATR inhibitor VE-821 sensitises ATM-deficient cells to olaparib and induces apoptosis. **a** A549-CRISPR-control cells (black bars) and A549-CRISPR-ATM A549 cells (grey bars) were treated with DMSO, 1 µM olaparib, 2 µM VE-821 or olaparib and VE-821 for 48-144 h as indicated, then samples were analysed using the trypan blue exclusion assay. The figure shows the mean with SEM of three independent experiments. Statistical significance was determined by one-way ANOVA. * Indicates *p*-value < 0.05 when compared to other treatment groups and DMSO control. # indicates p-value < 0.05 when compared to DMSO control only. **b** Percentages of A549-CRISPR-control (black bars) and A549-CRISPR-ATM cells (grey bars) undergoing apoptosis, as represented by the sub-G1 population. Shading and statistics are as in panel (**a**). **c** Percentages of cells undergoing apoptosis by annexin staining. Samples are as in panel (**b**). Shading and statistics are as in panel (**a**, **b**)

To answer this question, cells were treated with DMSO (control), olaparib (1 µM), VE-821 (2 µM) or a combination of olaparib and VE-821, then cells were assayed for viability and apoptosis as above. The combination of olaparib and VE-821 decreased viability in ATM-deficient cells after 96, 120 and 140 h (Fig. 6a). To determine whether ATM-deficient cells treated with the combination of olaparib and VE-821 were undergoing apoptosis, we assayed for sub-G1 DNA (Fig. 6b) and annexin staining (Fig. 6c). Significantly, in both experiments, apoptosis was observed in the A549-CRISPR-ATM cells but not control cells, suggesting that the combination of PARP inhibitor with inhibition of ATR is cytotoxic only in ATM-deficient cells (Fig. 6c).

To determine whether the addition of ATR inhibitor simply induced more DNA damage than PARP inhibitor alone, we asked whether ATR inhibitor would sensitise ATM-deficient cells to IR. A549-CRISPR-control and A549-CRISPR-ATM cells were either irradiated with 1, 2 or 4 Gy IR or treated with VE-821 then irradiated and analysed by trypan blue staining and flow cytometry for apoptosis. Addition of VE-821 did not decrease cell viability compared to IR or VE-821 alone and did not increase apoptosis under any conditions tested, suggesting that the enhanced cell death observed by the combination of olaparib and VE-821 in ATM-deficient cells is indeed due to inhibition of ATR and not to a general increase in DNA damage (Supplementary Fig. 10).

A recent report has shown that olaparib induces ROS in bladder cancer cells,^43^ therefore, we also determined whether ROS levels were elevated in our olaparib-treated ATM-deficient A549 cells using a fluorescence-based assay. Although hydrogen peroxide enhanced ROS production in this assay, neither ATM-deficient A549 cells or other cell line treated with olaparib showed elevated ROS levels (Supplementary Fig. 11), suggesting that ROS do not contribute to the DNA damage response in olaparib-treated A549 cells.

## Discussion

Using publicly available databases, we show that both *ATM* mutation and *ATM* deficiency (diploid versus amplified) are associated with olaparib sensitivity in a panel of human lung adenocarcinoma cell lines (Fig. 1). In contrast, sensitivity to talazoparib, a more potent PARP-trapping agent than olaparib,^65^ correlated with *ATM* expression and amplification status but not *ATM* mutational status (Fig. 1d). This may reflect the small number of ATM-deficient cell lines used in our analysis or subtle differences between *ATM* mutational status and PARP inhibitor sensitivity.

To empirically test whether olaparib can target ATM-deficient human cell lines, we used CRISPR/Cas9 to deplete ATM and DNA-PKcs from A549 cells, a human lung adenocarcinoma cell line, and used these cells to interrogate the mechanism by which lung adenocarcinoma cells respond to olaparib. Our results show that olaparib induces autophosphorylation of DNA-PKcs on serine 2056 and ATM on serine 1981 as well as H2AX serine 139 and p53 serine 15 phosphorylation, indicative of induction of a DNA damage response. Olaparib also induced upregulation of p21 and cyclin B1 and both phosphorylation and upregulation were more pronounced in ATM-deficient cells than in control or DNA-PKcs deficient cells. In addition, olaparib induced transient, reversible G2 arrest in ATM-deficient cells but not cell death. Since the G2/M checkpoint is only activated when the amount of DNA damage exceeds 10–20 DSBs per cell,^66^ we suggest that in ATM-proficient cells, the amount of olaparib-induced DNA damage is insufficient to induce the checkpoint, whereas when ATM is absent, unrepaired damage triggers checkpoint activation resulting in transient G2 arrest. Thus, in these ATM-deficient cells, olaparib alone was cytostatic but not cytotoxic. These observations could account for the decreased proliferation rate observed by us and others^38^ as well as the small colony size observed in the clonogenic survival assays (Supplementary Fig. 4). Our results also suggest that the type of assay used to measure cell viability and/or cell death may influence the interpretation of results using olaparib, as assays that measure mitochondrial cell death rather than viability or proliferative potential may not detect the transient, G2 arrest shown here in ATM-deficient cells.

Given that ATR is essential for the DNA damage-induced G2/M checkpoint,^61^ we hypothesised that inhibition of ATR would eliminate olaparib-induced G2 arrest, causing the cells to slip into mitosis, resulting in cell death. Indeed, the decrease in cyclin B1 expression observed in cells treated with both olaparib and VE-821 (Fig. 4), would support this idea. Moreover, addition of the ATR inhibitor VE-821 to olaparib-treated ATM-deficient A549 cells induced apoptosis, whereas ATM-proficient cells were relatively unaffected. Our findings are thus in line with other studies that have shown that the toxicity of ATR inhibitors is exacerbated by loss of ATM (discussed in ref. ^67^) but in our experiments, this only occurred in the additional presence of olaparib. Our studies are also reminiscent of the finding that ATR inhibition (with VE-821) is synergistic with both olaparib and talazoparib in cells deficient for Schlafen-11 (*SLFN11*) and that resistance to PARP inhibitors by Schlafen-11 inactivation can be overcome by ATR inhibition.^65^ Thus, we proposed that single-agent treatment PARP inhibitor treatment in patients with ATM-deficient tumours may be less efficacious than the combination of PARP inhibitor and ATR inhibitor and that future clinical trials may need to consider targeting ATM-deficient tumours with a PARP inhibitor combined with an ATR inhibitor. Interestingly, the ATR inhibitor AZD6738 is in clinical trials in combination with olaparib in triple-negative breast cancer,^67^ suggesting that the effects of PARP inhibitors in combination with ATR inhibitors could be tested in patients with ATM-deficient tumours.

Although frequently attributed solely to ATM, both DNA-PKcs and ATM contribute to DNA damage-induced phosphorylation of H2AX.^56^ Here, we show that olaparib induced H2AX phosphorylation is undetectable in CRISPR-DNA-PKcs cells and in ATM-deleted cells treated with the DNA-PK inhibitor NU7441, suggesting that DNA-PKcs is required for olaparib-induced phosphorylation of H2AX in these cells. Similarly, olaparib-induced phosphorylation of p53 and upregulation of p21 was reduced in DNA-PKcs-depleted cells. Like control cells, DNA-PKcs-deficient A549 cells did not arrest in G2 in response to olaparib and were not sensitive to olaparib in clonogenic assays. Together, these findings suggest that DNA-PKcs is important for DNA damage-induced phosphorylation of H2AX and initiating a DNA damage response in A549 cells, but that the level of DNA damage in these cells is below that needed to trigger the G2 checkpoint. In contrast, in the absence of ATM, additional DNA damage triggers the G2 checkpoint. However, this additional damage is still insufficient to induce cell death unless ATR is also inhibited (Supplementary Fig. 12). Thus, the three members of the PIKK family play important roles in responding to olaparib. Since olaparib-induced, DNA-PK-dependent phosphorylation of H2AX and p53 was enhanced in ATM-deficient cells, this suggests that the absence of ATM results in either more DNA damage and hence more signalling by DNA-PKcs or increased activity of DNA-PKcs.

In summary, our data suggest that olaparib induces DNA damage that leads to phosphorylation of ATM, DNA-PKcs, p53 and H2AX with consequent upregulation of p21 and cyclin B1. In control cells, this damage is repaired without triggering a G2/M checkpoint. In the absence of ATM, DNA-PK-dependent autophosphorylation (serine 2056), as well as phosphorylation of p53 (serine 15) and H2AX (serine 139), increased. The increase in DNA damage triggered by olaparib treatment and loss of ATM induces transient G2 arrest, but not apoptosis. Finally, we propose that inhibition of ATR by VE-821 causes the cells to proceed into mitosis with a corresponding increase in apoptotic cell death (Supplementary Fig. 12).

Our findings also confirm that loss of DNA-PKcs results in reduced ATM expression. We previously showed that M059J cells, which lack DNA-PKcs (then called p350)^68^ also have reduced levels of ATM protein expression.^50^ Similarly, siRNA depletion of DNA-PKcs resulted in reduced levels of ATM expression.^52^ Here we show that CRISPR/Cas9 deletion of DNA-PKcs also causes reduced ATM levels. The mechanism by which DNA-PKcs regulates ATM expression is not yet known, however, the isogenic cell lines generated and characterised in this study could be a valuable tool in understanding the interplay between DNA-PKcs and ATM in the DNA damage response. Interestingly, whereas both DNA-PKcs deleted and ATM deleted A549 cells were equally sensitive to IR, only ATM-deficient A549 cells were sensitive to olaparib. Thus, although CRISPR-deleted DNA-PKcs cells have only ~20% of the normal level of ATM, they still responded to olaparib in a similar manner to wild type cells. We speculate that even low levels of ATM expression may confer resistance to PARP inhibition, and further investigation to determine whether tumours with low or mutated ATM will be sensitive to olaparib plus or minus an ATR inhibitor.

It has recently been reported that ATM-null mouse thymocytes have mitochondrial DNA damage and enhanced levels of reactive oxygen species (ROS)^69^ and a recent report has shown that olaparib induces ROS in bladder cancer cells.^43^ However, we did not see evidence of elevated ROS in either ATM-deficient cells or A549 control cells treated with olaparib, suggesting that ROS do not contribute to olaparib induced toxicity in A549 cells. It is also worth noting that the bladder cancer cell line used in the previous study had mutant *TP53*^43^ which could also affect mechanism of action of olaparib. Indeed, we have previously reported that deletion of p53 in combination with inhibition or depletion of ATM enhanced sensitivity to olaparib in multiple cell types,^22–25^ however, cell death induced by ATR inhibitors is reported to be independent of p53 status (discussed in ref. ^67^) Whether p53 status will affect the response of ATM-deficient cells to combination ATR and PARP inhibition remains to be determined. However, since mutations in both *ATM* and *TP53* is rare in lung cancer cells (Supplementary Fig. 3) and other human tumours (1% of 400 tumours analysed),^70^ the strategy described here of targeting p53-proficient, ATM-deficient lung cancer cells with the combination of an ATR inhibitor with a PARP inhibitor may have clinical relevance in lung and other cancers.

## Supplementary information

Supplementary Material-CLEAN

## Data Availability

All original data is archived and stored at the Charbonneau Cancer Institute, University of Calgary, Alberta, Canada. Cell lines generated in this study (A549-CRISPR-control, A549-CRISPR-DNA-PKcs and A549-CRISPR-ATM) will be made available to other researchers upon request.

## References

[1] Farmer H, McCabe N, Lord CJ, Tutt AN, Johnson DA, Richardson TB (2005). Targeting the DNA repair defect in BRCA mutant cells as a therapeutic strategy. Nature.

[2] Bryant HE, Schultz N, Thomas HD, Parker KM, Flower D, Lopez E (2005). Specific killing of BRCA2-deficient tumours with inhibitors of poly(ADP-ribose) polymerase. Nature.

[3] Lord CJ, McDonald S, Swift S, Turner NC, Ashworth A (2008). A high-throughput RNA interference screen for DNA repair determinants of PARP inhibitor sensitivity. DNA Repair.

[4] Ashworth A, Lord CJ (2018). Synthetic lethal therapies for cancer: what’s next after PARP inhibitors?. Nat. Rev. Clin. Oncol..

[5] Thomas A, Murai J, Pommier Y (2018). The evolving landscape of predictive biomarkers of response to PARP inhibitors. J. Clin. Investig..

[6] Zimmer AS, Gillard M, Lipkowitz S, Lee JM (2018). Update on PARP inhibitors in breast cancer. Curr. Treat. options Oncol..

[7] McCabe N, Turner NC, Lord CJ, Kluzek K, Bialkowska A, Swift S (2006). Deficiency in the repair of DNA damage by homologous recombination and sensitivity to poly(ADP-ribose) polymerase inhibition. Cancer Res..

[8] Murai J, Huang SY, Das BB, Renaud A, Zhang Y, Doroshow JH (2012). Trapping of PARP1 and PARP2 by Clinical PARP Inhibitors. Cancer Res..

[9] Paull TT (2015). Mechanisms of ATM Activation. Annu. Rev. Biochem..

[10] Shiloh Yosef (2014). ATM: Expanding roles as a chief guardian of genome stability. Experimental Cell Research.

[11] Shiloh Y, Lederman HM (2017). Ataxia-telangiectasia (A-T): an emerging dimension of premature ageing. Ageing Res. Rev..

[12] Matsuoka S, Ballif BA, Smogorzewska A, McDonald ER, Hurov KE, Luo J (2007). ATM and ATR substrate analysis reveals extensive protein networks responsive to DNA damage. Science.

[13] Bennetzen MV, Larsen DH, Bunkenborg J, Bartek J, Lukas J, Andersen JS (2010). Site-specific phosphorylation dynamics of the nuclear proteome during the DNA damage response. Mol. Cell Proteomics.

[14] Goodarzi AA, Noon AT, Deckbar D, Ziv Y, Shiloh Y, Lobrich M (2008). ATM signaling facilitates repair of DNA double-strand breaks associated with heterochromatin. Mol. Cell.

[15] McKinnon PJ (2001). Ataxia telangiectasia: new neurons and ATM. Trends Mol. Med.

[16] Schaffner C, Idler I, Stilgenbauer S, Dohner H, Lichter P (2000). Mantle cell lymphoma is characterized by inactivation of the ATM gene. Proc. Natl Acad. Sci. USA.

[17] Greiner TC, Dasgupta C, Ho VV, Weisenburger DD, Smith LM, Lynch JC (2006). Mutation and genomic deletion status of ataxia telangiectasia mutated (ATM) and p53 confer specific gene expression profiles in mantle cell lymphoma. Proc. Natl Acad. Sci. USA.

[18] Seshagiri S, Stawiski EW, Durinck S, Modrusan Z, Storm EE, Conboy CB (2012). Recurrent R-spondin fusions in colon cancer. Nature.

[19] Ding L, Getz G, Wheeler DA, Mardis ER, McLellan MD, Cibulskis K (2008). Somatic mutations affect key pathways in lung adenocarcinoma. Nature.

[20] Mateo J, Boysen G, Barbieri CE, Bryant HE, Castro E, Nelson PS (2017). DNA repair in prostate cancer: biology and clinical implications. Eur. Urol..

[21] Choi M, Kipps T, Kurzrock R (2016). ATM Mutations in Cancer: Therapeutic Implications. Mol. Cancer Ther..

[22] Williamson CT, Kubota E, Hamill JD, Klimowicz A, Ye R,, Muzik H (2012). Enhanced cytotoxicity of PARP inhibition in mantle cell lymphoma harbouring mutations in both ATM andp53. EMBO Mol. Med..

[23] Williamson CT, Muzik H, Turhan AG, Zamo A, O’Connor MJ, Bebb DG (2010). ATM deficiency sensitizes mantle cell lymphoma cells to poly(ADP-ribose) polymerase-1 inhibitors. Mol. cancer Ther..

[24] Wang C, Jette N, Moussienko D, Bebb DG, Lees-Miller SP (2017). ATM-Deficient colorectal cancer cells are sensitive to the PARP inhibitor olaparib. Transl. Oncol..

[25] Kubota E, Williamson CT, Ye R, Elegbede A, Peterson L, Lees-Miller SP (2014). Low ATM protein expression and depletion of p53 correlates with olaparib sensitivity in gastric cancer cell lines. Cell Cycle.

[26] Petersen LF, Klimowicz AC, Otsuka S, Elegbede AA, Petrillo SK, Williamson T (2017). Loss of tumour-specific ATM protein expression is an independent prognostic factor in early resected NSCLC. Oncotarget..

[27] Gao J, Aksoy BA, Dogrusoz U, Dresdner G, Gross B, Sumer SO (2013). Integrative analysis of complex cancer genomics and clinical profiles using the cBioPortal. Sci. Signal..

[28] Cerami E, Gao J, Dogrusoz U, Gross BE, Sumer SO, Aksoy BA (2012). The cBio cancer genomics portal: an open platform for exploring multidimensional cancer genomics data. Cancer Discov..

[29] Villaruz L. C., Jones H., Dacic S., Abberbock S., Kurland B. F., Stabile L. P. et al. ATM protein is deficient in over 40% of lung adenocarcinomas. *Oncotarget.* 2016; e-pub ahead of print 2016/06/04; 10.18632/oncotarget.9757. 10.18632/oncotarget.9757 PMC5295384 27259260

[30] Torok Jordan A., Oh Patrick, Castle Katherine D., Reinsvold Michael, Ma Yan, Luo Lixia, Lee Chang-Lung, Kirsch David G. (2018). Deletion of Atm in Tumor but not Endothelial Cells Improves Radiation Response in a Primary Mouse Model of Lung Adenocarcinoma. Cancer Research.

[31] Schmitt A, Knittel G, Welcker D, Yang TP, George J, Nowak M (2017). ATM Deficiency is associated with sensitivity to PARP1- and ATR Inhibitors in lung adenocarcinoma. Cancer Res..

[32] Langelier MF, Eisemann T, Riccio AA, Pascal JM (2018). PARP family enzymes: regulation and catalysis of the poly(ADP-ribose) posttranslational modification. Curr. Opin. Struct. Biol..

[33] Schreiber V, Dantzer F, Ame JC, de Murcia G (2006). Poly(ADP-ribose): novel functions for an old molecule. Nat. Rev. Mol. cell Biol..

[34] Mansour WY, Rhein T, Dahm-Daphi J (2010). The alternative end-joining pathway for repair of DNA double-strand breaks requires PARP1 but is not dependent upon microhomologies. Nucleic acids Res..

[35] Fisher AE, Hochegger H, Takeda S, Caldecott KW (2007). Poly(ADP-ribose) polymerase 1 accelerates single-strand break repair in concert with poly(ADP-ribose) glycohydrolase. Mol. Cell. Biol..

[36] Gottipati P, Vischioni B, Schultz N, Solomons J, Bryant HE, Djureinovic T (2010). Poly(ADP-ribose) polymerase is hyperactivated in homologous recombination-defective cells. Cancer Res..

[37] Strom CE, Johansson F, Uhlen M, Szigyarto CA, Erixon K, Helleday T (2011). Poly (ADP-ribose) polymerase (PARP) is not involved in base excision repair but PARP inhibition traps a single-strand intermediate. Nucleic acids Res..

[38] Maya-Mendoza Apolinar, Moudry Pavel, Merchut-Maya Joanna Maria, Lee MyungHee, Strauss Robert, Bartek Jiri (2018). High speed of fork progression induces DNA replication stress and genomic instability. Nature.

[39] Hanzlikova H, Kalasova I, Demin AA, Pennicott LE, Cihlarova Z, Caldecott KW (2018). The importance of poly(ADP-Ribose) polymerase as a sensor of unligated Okazaki fragments during dna replication. Mol. Cell.

[40] Weston VJ, Oldreive CE, Skowronska A, Oscier DG, Pratt G, Dyer MJ (2010). The PARP inhibitor olaparib induces significant killing of ATM-deficient lymphoid tumor cells in vitro and in vivo. Blood.

[41] Dale Rein I, Stokke C, Jalal M, Myklebust JH, Patzke S, Stokke T (2015). New distinct compartments in the G2 phase of the cell cycle defined by the levels of gammaH2AX. Cell Cycle.

[42] Dale Rein I, Solberg Landsverk K, Micci F, Patzke S, Stokke T (2015). Replication-induced DNA damage after PARP inhibition causes G2 delay, and cell line-dependent apoptosis, necrosis and multinucleation. Cell Cycle.

[43] Liu Q, Gheorghiu L, Drumm M, Clayman R, Eidelman A, Wszolek MF (2018). PARP-1 inhibition with or without ionizing radiation confers reactive oxygen species-mediated cytotoxicity preferentially to cancer cells with mutant TP53. Oncogene.

[44] Lee KJ, Lin YF, Chou HY, Yajima H, Fattah KR, Lee SC (2011). Involvement of DNA-dependent protein kinase in normal cell cycle progression through mitosis. J. Biol. Chem..

[45] Meek K, Douglas P, Cui X, Ding Q, Lees-Miller SP (2007). trans Autophosphorylation at DNA-dependent protein kinase’s two major autophosphorylation site clusters facilitates end processing but not end joining. Mol. Cell. Biol..

[46] Reaper PM, Griffiths MR, Long JM, Charrier JD, Maccormick S, Charlton PA (2011). Selective killing of ATM- or p53-deficient cancer cells through inhibition of ATR. Nat. Chem. Biol..

[47] Greenman CD, Bignell G, Butler A, Edkins S, Hinton J, Beare D (2010). PICNIC: an algorithm to predict absolute allelic copy number variation with microarray cancer data. Biostat. (Oxf., Engl.).

[48] Durinck S, Spellman PT, Birney E, Huber W (2009). Mapping identifiers for the integration of genomic datasets with the R/Bioconductor package biomaRt. Nat. Protoc..

[49] H W. (2009). ggplot2: elegant graphics for data analysis..

[50] Chan DW, Gately DP, Urban S, Galloway AM, Lees-Miller SP, Yen T (1998). Lack of correlation between ATM protein expression and tumour cell radiosensitivity. Int. J. Radiat. Biol..

[51] Goodwin JF, Kothari V, Drake JM, Zhao S, Dylgjeri E, Dean JL (2015). DNA-PKcs-mediated transcriptional regulation drives prostate cancer progression and metastasis. Cancer cell.

[52] Peng Y, Woods RG, Beamish H, Ye R, Lees-Miller SP, Lavin MF (2005). Deficiency in the catalytic subunit of DNA-dependent protein kinase causes down-regulation of ATM. Cancer Res..

[53] Bakkenist CJ, Kastan MB (2003). DNA damage activates ATM through intermolecular autophosphorylation and dimer dissociation. Nature.

[54] Ciccia A, Elledge SJ (2010). The DNA damage response: making it safe to play with knives. Mol. Cell.

[55] Bonner WM, Redon CE, Dickey JS, Nakamura AJ, Sedelnikova OA, Solier S (2008). GammaH2AX and cancer. Nat. Rev. Cancer.

[56] Stiff T, O’Driscoll M, Rief N, Iwabuchi K, Lobrich M, Jeggo PA (2004). ATM and DNA-PK function redundantly to phosphorylate H2AX after exposure to ionizing radiation. Cancer Res..

[57] Solier S, Kohn KW, Scroggins B, Xu W, Trepel J, Neckers L (2012). Heat shock protein 90alpha (HSP90alpha), a substrate and chaperone of DNA-PK necessary for the apoptotic response. Proc. Natl. Acad. Sci. USA.

[58] Solier S, Pommier Y (2009). The apoptotic ring: a novel entity with phosphorylated histones H2AX and H2B and activated DNA damage response kinases. Cell Cycle.

[59] Moeglin Eric, Desplancq Dominique, Conic Sascha, Oulad-Abdelghani Mustapha, Stoessel Audrey, Chiper Manuela, Vigneron Marc, Didier Pascal, Tora Laszlo, Weiss Etienne (2019). Uniform Widespread Nuclear Phosphorylation of Histone H2AX Is an Indicator of Lethal DNA Replication Stress. Cancers.

[60] Leahy JJ, Golding BT, Griffin RJ, Hardcastle IR, Richardson C, Rigoreau L (2004). Identification of a highly potent and selective DNA-dependent protein kinase (DNA-PK) inhibitor (NU7441) by screening of chromenone libraries. Bioorg. Med. Chem. Lett..

[61] Shaltiel IA, Krenning L, Bruinsma W, Medema RH (2015). The same, only different - DNA damage checkpoints and their reversal throughout the cell cycle. J. cell Sci..

[62] Huntoon CJ, Flatten KS, Wahner Hendrickson AE, Huehls AM, Sutor SL, Kaufmann SH (2013). ATR inhibition broadly sensitizes ovarian cancer cells to chemotherapy independent of BRCA status. Cancer Res..

[63] Peasland A, Wang LZ, Rowling E, Kyle S, Chen T, Hopkins A (2011). Identificationand evaluation of a potent novel ATR inhibitor, NU6027, in breast and ovariancancer cell lines. Br J. Cancer.

[64] Rundle Stuart, Bradbury Alice, Drew Yvette, Curtin Nicola (2017). Targeting the ATR-CHK1 Axis in Cancer Therapy. Cancers.

[65] Murai J, Feng Y, Yu GK, Ru Y, Tang SW, Shen Y (2016). Resistance to PARP inhibitors by SLFN11 inactivation can be overcome by ATR inhibition. Oncotarget.

[66] Lobrich M, Jeggo PA (2007). The impact of a negligent G2/M checkpoint on genomic instability and cancer induction. Nat. Rev. Cancer.

[67] Lecona E, Fernandez-Capetillo O (2018). Targeting ATR in cancer. Nat. Rev. Cancer.

[68] Lees-Miller SP, Godbout R, Chan DW, Weinfeld M, Day RS, Barron GM (1995). Absence of p350 subunit of DNA-activated protein kinase from a radiosensitive human cell line. Sci. (New Y., NY).

[69] Valentin-Vega YA, Maclean KH, Tait-Mulder J, Milasta S, Steeves M, Dorsey FC (2012). Mitochondrial dysfunction in ataxia-telangiectasia. Blood.

[70] Jiang H, Reinhardt HC, Bartkova J, Tommiska J, Blomqvist C, Nevanlinna H (2009). The combined status of ATM and p53 link tumor development with therapeutic response. Genes Dev..

