# Correction: Combined poly-ADP ribose polymerase and ataxia-telangiectasia mutated/Rad3-related inhibition targets ataxia-telangiectasia mutated-deficient lung cancer cells

**DOI:** 10.1038/s41416-020-0841-7

**Published:** 2020-04-17

**Authors:** Nicholas R. Jette, Suraj Radhamani, Greydon Arthur, Ruiqiong Ye, Siddhartha Goutam, Anthony Bolyos, Lars F. Petersen, Pinaki Bose, D. Gwyn Bebb, Susan P. Lees-Miller

**Affiliations:** 1 Departments of Biochemistry and Molecular Biology, Robson DNA Science Centre and Charbonneau Cancer Institute, Cumming School of Medicine, University of Calgary, 3330 Hospital Drive NW, Calgary, Alberta T2N 1N4 Canada; 2 Department Oncology, Robson DNA Science Centre and Charbonneau Cancer Institute, Cumming School of Medicine, University of Calgary, 3330 Hospital Drive NW, Calgary, Alberta T2N 1N4 Canada

**Keywords:** Cancer, Non-small-cell lung cancer, Lung cancer

Correction to: *British Journal of Cancer* 10.1038/s41416-019-0565-8, published online 4 September 2019

This Article was originally published under Nature Research’s License to Publish, but has now been made available under a CC BY 4.0 license.

This has now been corrected in both the PDF and HTML versions of the Article.

