# Supplementary material for: Combined poly-ADP ribose polymerase and ataxia-telangiectasia mutated/Rad3-related inhibition targets ataxia-telangiectasia mutated-deficient lung cancer cells
Source: Br J Cancer. 2019 Sep 4;121(7):600–10. doi: 10.1038/s41416-019-0565-8 (PMC6889280; doi:10.1038/s41416-019-0565-8)
Supplement: Supplementary file 1 — Supplementary Material-CLEAN [file 41416_2019_565_MOESM1_ESM.pdf]

SUPPLEMENTARY MATERIAL

Contains

1. Supplementary Methods

Generation of CRISPR/Cas9 control, DNA-PKcs and ATM cells

SDS PAGE and western blot

Clonogenic survival assays

Colony size in clonogenic assays

Cell cycle distribution by propidium iodide staining

Annexin staining for apoptosis

Trypan blue exclusion assays

γ–H2AX foci analysis

ROS assays

2. Supplementary Tables

Supplementary Table 1:

Boxplot showing the correlation between *ATM* gene amplification and expression levels in 61 lung adenocarcinoma cell lines from the GDSC project.

Supplementary Table 2:

Suppliers and catalogue numbers for antibodies used in this study.

3. Supplementary Figures.

Supplementary Figure 1:

Boxplot showing the correlation between *ATM* gene amplification and expression levels in 61 lung adenocarcinoma cell lines from the GDSC project.

Supplementary Figure 2:

Boxplot comparing olaparib IC_{50} levels between top and bottom quartile *ATM* expressing lung adenocarcinoma cell lines from the GDSC project.

Supplementary Figure 3:

Oncoprint showing the mutual exclusivity of *ATM* and *TP53* gene mutations in TCGA lung adenocarcinoma (LUAD) patients.

Supplementary Figure 4:

Olaparib treatment results in smaller colony size in ATM-deficient cells than control cells.

Supplementary Figure 5:

Olaparib reduces cell proliferation in both control and ATM-deficient A549 cells.

Supplementary Figure 6:

Western blots of two additional experiments described and quantitated in Figure 2.

Supplementary Figure 7:

γ-H2AX foci analysis of olaparib-treated A549-CRISPR-control, A549-CRISPR-DNA-PKcs and A549-CRISPR-ATM cells.

Supplementary Figure 8:

Western blots of two additional experiments described and quantitated in Figure 3.

Supplementary Figure 9:

ATM-deficient A549 cells do not undergo apoptosis when treated with olaparib.

Supplementary Figure 10:

VE-821 does not enhance sensitivity of ATM-deficient A549 cells to ionizing radiation.

Supplementary Figure 11:

Neither loss of ATM nor addition of olaparib induces reactive oxygen species in A549 lung adenocarcinoma cells.

Supplementary Figure 12:

Model showing olaparib induces reversible G2 arrest in A549-CRISPR-ATM cells while the combination of olaparib plus VE-821 induces cell death.

1. SUPPLEMENTARY METHODS

CRISPR/Cas9 deletion of DNA-PKcs and ATM.

Deletion of DNA-PKcs and ATM from A549 cells was carried out by Dr. Yaping Yu at the Center for Genome Engineering, Cumming School of Medicine, University of Calgary.

For CRISPR deletion of DNA-PKcs from A549 cells, the pSpCas9(BB)-2A-GFP (pX458) vector (a gift from Feng Zhang; Addgene plasmid # 48138; http://n2t.net/addgene:48138 RRID:Addgene_48138) was used.
The short guide sequence to target DNA-PKcs exon thirty-one was:
5'-TGTAGCACTCCAACGCGGCC- 3'.
CRISPR deletion of ATM in A549 cells was carried out using the pSpCas9(BB)-2A-GFP (pX458) vector and a short guide sequence targeting ATM exon nine,
5'-TACGTTCCCCATGTCGCTGT-3'.

All short guide oligonucleotides were synthesized, annealed and cloned into pX458 vector and were confirmed by DNA sequencing at DNA laboratory, University of Calgary.

Five (5) µg DNA plasmid (pX458 containing DNA-PKcs or ATM short guide RNA sequence) was transfected into A549 cells respectively using Lipofectamine 2000 (Invitrogen) according to the manufacturer's instructions.

48h after transfection (A549 CRISPR-DNA-PKcs and ATM cells), cells were harvested and genomic DNA was isolated using the KAPA Express Extract Kit (Kapa Biosystems) according to the manufacturer's instructions. Genomic DNA fragments of DNA-PKcs and ATM around the short guide RNA site were amplified by PCR.

The primers used to amplify the genomic region of DNA-PKcs exon thirty-one were:
Forward: 5'-CAGCCCACCCTCTTGTACC-3',
Reverse: 5'-TGAAGTGTGAAGAATGGAACCC-3'.

The primers for amplification of genomic region of ATM exon nine were:
Forward: 5'-AGGCTACAGATTGCAACCA-3',
Reverse: 5'-CATGAAGGTCTGCAGGCTGA-3'.

PCR products were used for Surveyor nuclease mutation detection assays using the SURVEYOR Mutation Detection Kit (Transgenomic) according to manufacturer's protocol.

After transfection, GFP containing cells were sorted into 96 wells plate by Flow Cytometry, at the Flow Cytometry Facility, University of Calgary. Single cells were then expanded for further analysis (i.e., Western blot and DNA sequencing).

All CRISPR gene knockout clones were screened by Western blot using anti-ATM (Millipore) or anti-DNA-PKcs (in house) antibodies. The membrane was also probed with Mre11 antibody (Novus) as a loading control (see SDS PAGE and Western blot).

For DNA sequencing confirmation of DNA-PKcs and ATM knockout clones, DNA fragments of DNA-PKcs knockout clone and ATM knockout clone around the short guide RNA site were amplified by PCR. PCR products were subcloned into pEGFP-C2 vector (Clontech) and plasmid DNA from individual clones were sent for Sanger DNA sequencing at DNA laboratory, University of Calgary to confirm all indela.

Generation of CRIPSR control cells: pSpCas9(BB)-2A-GFP (pX458) vector was transfected into A549 cells with Lipofectamine 2000 according to the manufacturer's instructions. 48h later, GFP containing cells were separated by flow cytometry.

Q-PCR: Total RNA was isolated using RNeasy Mini Kit (QIAGEN) according to the manufacturer's recommended condition and RNA was reverse transcribed using a QuantiTect Reverse Transcription Kit (QIAGEN). Gene-specific primer sequences are available from the authors upon request. Real-time PCR was carried out in triplicate using PowerUp™ SYBR® Green Master Mix A25777 (Thermofisher) on a Roche LightCycler 96 system using a standard protocol. GAPDH were used as endogenous normalization controls. Relative fold changes were determined using the comparative threshold (CT) method.

SDS PAGE and western blot

Whole cell extracts were generated by NETN lysis and protein concentrations determined using the Biorad Detergent Compatible Protein Assay with BSA as standard as described previously [1]. Fifty (50) µg total protein was run on SDS PAGE, proteins were transferred to nitrocellulose and developed using ECL reagent and X-ray film as described previously. H2AX was extracted from the NETN pellet by heating in 1% SDS as described previously [1]. Suppliers of antibodies are shown in Supplementary Table 2.

Clonogenic survival assays and irradiation

Clonogenic survival assays were carried out as described previously [2]. Briefly, cells were seeded in 6 cm dishes and after 24-hours, either irradiated in complete media using a ^{137}Cs source Gammacell 1000 Elite tissue irradiator (MDS Nordion) at 2.9 Gy/min or incubated with olaparib (made up in DMSO) at the concentrations indicated. An equal volume of DMSO was added to control plates. Drugs were left on for the entire experiment. After 10 days, cells were fixed, stained with crystal violet and counted using a Col-Counter (Oxford Instruments).

Counting of colony size from clonogenic survival assays

Following staining with crystal violet and counting the colonies for survival fraction, the plates were also used for measuring the colony radius. A minimum colony radius of 0.02 mm and a maximum colony radius of 5.00 mm was used as the measuring criteria. Also, the minimum colony density of 0.02 optical density (O.D.) was set for measuring. Colonies that fell between 0.02 and 5 mm were measured and graphed as a density plot. Statistical

significance was determined using a one-way ANOVA, and p values less than 0.05 (represented by *) were considered statistically significant.

Cell Cycle distribution by propidium iodide (PI) staining

Following treatment, cells were centrifuged (5 minutes, 4°C, 2,500 g) and media was removed. Cells were washed 1 X in 2 ml 1X PBS then fixed by re-suspending in 0.5 mL 1% (w/v) NaCl solution, and a 95% ethanol solution was then added dropwise to the cells (to the amount of 0.5 mL) while vortexing. Cells were then incubated for 30 minutes at room temperature (RT) and then stored at 4°C for up to one week. Immediately prior to analysis, cells were centrifuged (5 minutes, 4°C, 2,500g), the NaCl/Ethanol solution was removed and cells were resuspended in a 1 mL PI (5 µg) in PBS solution containing 1 mg/mL RNase (Sigma, R6513). Cells were then incubated in the dark (30 minutes, 37°C), then analyzed by flow cytometry by the University of Calgary Flow Cytometry facility.

Annexin staining for apoptosis

Annexin staining was carried out using a Dead Cell Apoptosis Kit (ThermoFisher Scientific Cat#V13241) according to the manufacturer's recommended conditions. Following treatment and incubation, cells were harvested and washed with PBS. After washing, cells were resuspended in 100 µL 1X annexin-binding buffer containing 100 µg/mL of propidium iodide and 5 µL Alexa Fluor® 488 annexin V. Cells were then incubated for another 15 minutes at RT. 400 µL 1X annexin-binding buffer was added and cells were gently resuspended. Samples were kept on ice and analyzed

immediately by flow cytometry as above. Staurosporine (Sigma-Aldrich S5921) was dissolved in DMSO and added to cells at a final concentration of 1 μM as a positive control.

Trypan Blue Exclusion assay

Following treatment, cells were harvested with 1X PBS-trypsin (0.05% Trypsin-EDTA, Gibco), pelleted by centrifugation and washed twice in PBS. Pellets were resuspended in 1 mL of fresh media and 10 μL of cell suspension was mixed with 10 μL Trypan Blue dye (0.40% (v/v)) (Bio-Rad) and pipetted into dual chamber counting slides (Bio-Rad) and cells were counted using a TC-20 automated cell counter (Bio-Rad). All experiments were carried out in duplicate and the results from three separate experiments are shown.

γ–H2AX foci analysis

A549-CRISPR-control, A549-CRISPR-ATM and A549-CRISPR-DNA-PKcs cells were seeded on poly-D-lysine coated coverslips and were permitted to adhere for 24 hours. After addition of olaparib for the indicated time, media was removed from the plates containing the cover slips and the coverslips were washed with 1 mL 1 X PBS. After washing, cells were fixed by adding 0.1 mL of 3.7% (v/v) paraformaldehyde and the coverslips were incubated for 10 minutes. Coverslips were then washed with 1 mL 1xPBS. Following washing, 0.1 mL of 0.2% Triton X-100 was added to the coverslip and samples were incubated for 10 minutes to permeabilize the cells. Coverslips were then washed with 1 mL of 1 X PBS. After this, 0.1 mL of 2% (w/v) BSA in 1 X PBS was added

to the coverslips and they were incubated at RT for 30 minutes. Primary antibody to γ-H2AX mouse (Abcam ab26350) 1:500 dilution in 1% (w/v) BSA in PBS was added and coverslips were incubated for 1 hour. Coverslips were then washed six times with 0.5% Tween 20 in PBS. After washing, 0.1 mL of secondary antibody Alexa 488 conjugated goat anti-mouse was added to each coverslip and samples were incubated for 30 minutes in the dark at RT, thereafter 0.1 mL of 0.1 µg/mL DAPI in PBS was added to the cells and they were incubated for a further 10 minutes. Coverslips were then washed dropwise with 0.1 mL water, which was aspirated immediately. Following this, approximately 0.1 mL of 62 Fluoromount-G (Thermo Fisher) was added to each coverslip. Coverslips were then placed on microscope slides and permitted to harden in the dark overnight. Fluorescence microscope images were captured as described below and image quantification was performed using ImageJ.

Images were acquired at RT on a Nikon Ti Eclipse widefield microscope equipped with a Hamamatsu Orca flash 4.0 v2 sCMOS 16-bit camera using NIS-Elements AR v5.00.00 64-bit software. Images for counting were captured using a 40x Plan Apo λ 0.95 numerical aperture (NA) objective. Images for presentation were captured using a 60x Plan Apo λ 1.4 NA oil objective. Images captured with the same objective were acquired using identical light power and exposure time settings, which were set to optimize visualization of the brightest samples, in this case, A549-CRISPR-ATM cells treated with olaparib for 1 day. Scale bars representing 10 µm are shown in the lower right of each image.

H2DCFDA assays

A stock solution of 20 mM H2DCFDA (Life Technologies, D399) in DMSO was freshly prepared every time just prior to assay to prevent oxidation. DMEM containing phenol red was removed from each plate and saved for later use. Plates were washed once with PBS. DMEM without phenol red containing 20 μM of H2DCFDA was added to each dish and dishes were further incubated for 1 hour. After incubation, media was removed and plates were washed once with PBS. Plates were replaced with DMEM phenol red media which was removed earlier and further incubated for 20 minutes. For a positive control, 500 μM H_2O_2 was added and incubated for 20 minutes at room temperature. Following treatment, media was removed and cells were washed once with PBS. Cells were then trypsinized and harvested with ice cold PBS, centrifuged for 5 minutes, 2400 rpm at 4^oC. The supernatant was removed by aspiration and the cells were resuspended in PBS containing 0.0025% of trypan blue to quench the extracellular dye. Samples were analyzed by flow cytometry at excitation/emission wavelengths of 492–495/517–527 nm.

2. SUPPLEMENTARY TABLES

Supplementary Table 1: ATM copy number and mutation status for 61 LUAD cell lines from GDSC.

Cell_line	COSMIC_ID	TCGA_code	ATM_Copy Num	ATM_MUT	olaparib_ln_IC50	ATM_mRNA_expression
HCC-44	1240145	LUAD	3	NO	2.334	5.456
HCC-827	1240146	LUAD	3	NO	3.838	5.004
NCI-H1944	1240185	LUAD	2	NO	4.087	5.067
NCI-H2023	1240187	LUAD	2	NO	2.630	5.332
NCI-H3122	1240190	LUAD	3	NO	3.925	5.055
PC-3 [JPC-3]	1240202	LUAD	5	YES	4.533	5.667
H3255	1247873	LUAD	1	NO	4.119	4.151
201T	1287381	LUAD	2	NO	4.436	4.954
HCC-78	1290908	LUAD	6	NO	3.422	5.381
NCI-H1435	1298347	LUAD	1	NO	3.533	4.787
NCI-H1568	1298348	LUAD	3	YES	3.822	5.030
NCI-H1781	1298350	LUAD	2	NO	3.965	4.853
RERF-LC-KJ	1298537	LUAD	3	NO	4.656	6.018
EMC-BAC-1	1503369	LUAD	3	NO	4.266	4.989
EMC-BAC-2	1503370	LUAD	2	NO	2.777	5.640
NCI-H1395	684681	LUAD	2	YES	3.572	4.703
Calu-3	687777	LUAD	2	NO	4.486	4.441
NCI-H1623	687798	LUAD	3	NO	3.916	4.572
NCI-H1648	687799	LUAD	3	NO	4.187	5.127
NCI-H1693	687802	LUAD	4	NO	3.516	5.601
NCI-H1838	687807	LUAD	6	YES	5.220	6.262

NCI-H2085	687812	LUAD	3	NO	3.515	5.713
NCI-H2342	687819	LUAD	4	NO	3.725	4.482
NCI-H2347	687820	LUAD	4	NO	3.658	4.492
NCI-H2405	687821	LUAD	4	NO	4.166	5.503
VMRC-LCD	713869	LUAD	2	NO	3.586	5.135
NCI-H2030	722045	LUAD	2	NO	3.384	5.950
NCI-H2122	722046	LUAD	2	NO	3.245	4.929
NCI-H1734	722058	LUAD	3	YES	2.677	5.587
NCI-H650	722066	LUAD	7	YES	4.143	6.116
NCI-H2087	724834	LUAD	4	YES	3.842	5.701
Calu-6	724859	LUAD	2	NO	4.348	4.919
NCI-H1355	724866	LUAD	3	NO	4.456	5.580
NCI-H1792	724868	LUAD	2	NO	3.496	6.060
NCI-H2009	724873	LUAD	4	NO	3.450	6.121
NCI-H2291	724874	LUAD	3	NO	5.094	4.625
SW1573	724878	LUAD	3	NO	3.810	6.191
LXF-289	753592	LUAD	3	YES	3.198	5.731
NCI-H1563	753600	LUAD	4	NO	5.080	5.212
PC-14	753608	LUAD	3	NO	2.882	4.798
NCI-H23	905942	LUAD	2	YES	2.684	5.536
NCI-H522	905944	LUAD	3	NO	4.551	6.393
A549	905949	LUAD	2	NO	2.527	5.334
NCI-H322M	905967	LUAD	3	NO	5.301	6.004
EKVX	905970	LUAD	13	NO	6.035	8.457
HOP-62	905972	LUAD	2	NO	2.365	5.525
ABC-1	906791	LUAD	3	NO	3.920	5.376
COR-L105	906805	LUAD	1	NO	3.152	4.995

LC-2-ad	907786	LUAD	4	NO	2.965	5.983
NCI-H441	908460	LUAD	3	NO	4.632	5.270
NCI-H1793	908463	LUAD	3	NO	5.299	4.965
NCI-H358	908465	LUAD	3	NO	4.614	5.816
NCI-H1573	908472	LUAD	1	NO	4.858	4.958
NCI-H1666	908473	LUAD	2	YES	2.969	4.602
NCI-H1755	908475	LUAD	3	NO	4.842	5.393
NCI-H1993	908476	LUAD	13	NO	4.983	7.030
SK-LU-1	909721	LUAD	5	NO	2.884	4.833
NCI-H838	910399	LUAD	2	NO	3.805	5.462
NCI-H1651	910900	LUAD	3	NO	1.928	5.234
RERF-LCMS	910931	LUAD	4	NO	2.573	5.519
NCI-H1975	924244	LUAD	3	NO	4.475	5.758

Supplementary Table 2: Antibodies used in this study

Antibody	Supplier	Catalogue number
ATM	Upstate	05-513
ATM p1981	Epitomics	2152-1
ATR	Millipore	09-070
Chk1	Cell Signaling	2345
Chk1 pS345	Cell Signaling	2341
Chk2	Novus	NBP 1-47695
Chk2 pT68	Cell Signaling	2661
Cyclin B1	Santa Cruz	Sc-7393
DNA-PKcs	In house	n/a
DNA-PKcs p2056	Abcam	Ab18192
Histone H2AX pS139 (western blot)	Millipore	05-636
Histone H2AX pS139 (immunofluorescence)	Abcam	Ab26350
Histone H3	Abcam	Ab1791
Histone H2AX	Abcam	Ab11175
Ku80	Abcam	Ab33242
mTOR	Millipore	05-1564
P21	Santa Cruz	Sc-756
P53	Santa Cruz	Sc-126

P53 pS15	Cell Signaling	9284

SUPPLEMENTARY FIGURES AND FIGURE LEGENDS

Supplementary Figure 1: Boxplot showing the correlation between *ATM* gene amplification and ATM mRNA expression levels in 61 lung adenocarcinoma cell lines from the GDSC project. Cell lines containing > 3 copies of the *ATM* gene were classified as "ATM amplified".

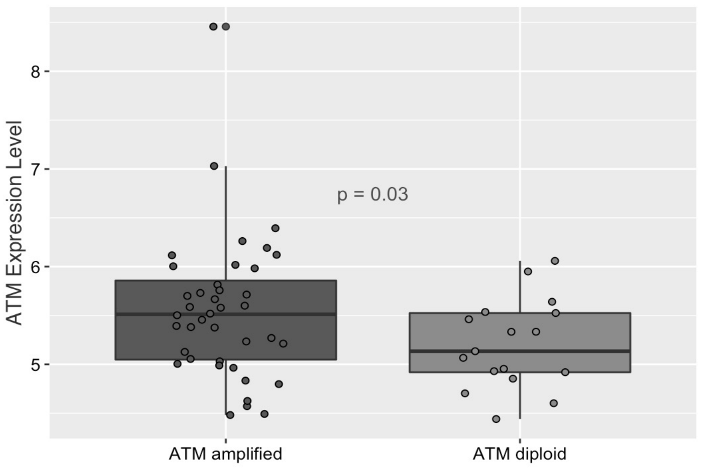

Supplementary Figure 2: Boxplot comparing olaparib IC_{50} levels between top and bottom quartile *ATM* expressing lung adenocarcinoma cell lines from the GDSC project.

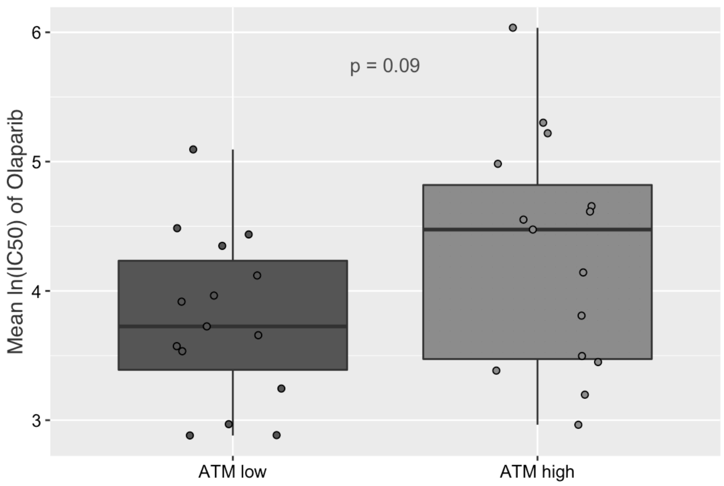

Supplementary Figure 3: Oncoprint showing the mutual exclusivity of *ATM* and *TP53* gene mutations in TCGA lung adenocarcinoma (LUAD) patients. Each grey bar represents a single LUAD patient. Data was downloaded from www.cbioportal.org.

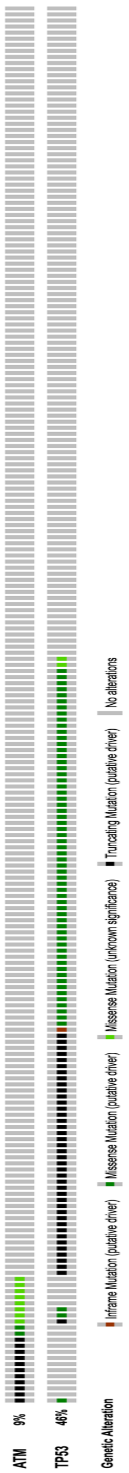

Supplementary Figure 4: Olaparib treatment results in smaller colony size in ATM-deficient cells than control cells. Clonogenic survival assays were carried out on 6 cm plates as described in Materials and Methods. The average radius was then measured using ColCount (see Supplementary methods for details). At least 300 colonies were counted per sample. Median with scatter is shown. To compare the relative colony sizes, the sizes of colonies in olaparib-treated cells were compared to those in the DMSO control. In each case, the median colony radius decreased in the presence of olaparib. Median values are shown below in the graph and the table below. Statistical significance was calculated using one-way ANOVA with Tukey's multiple comparison test. P values less than 0.05 were considered statistically significant. *** indicated $p < 0.001$. The top panel shows the actual values and the table below, the median values. Please see next page.

Supplementary Figure 4

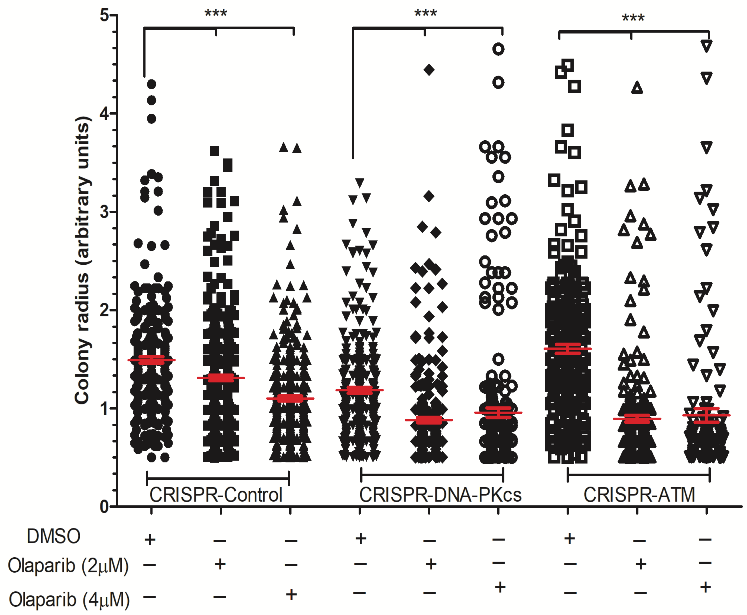

	CRISPR-control			CRISPR-DNA-PKcs			CRISPR-ATM		
	**DMSO**	**2 µM olaparib**	**4 µM olaparib**	**DMSO**	**2 µM olaparib**	**4 µM olaparib**	**DMSO**	**2 µM olaparib**	**4 µM olaparib**
**Colonies counted**	306	343	368	296	304	237	238	241	123
**Median**	1.485	1.233	1.031	1.085	0.7398	0.6975	1.537	0.7500	0.6287

Supplementary Figure 5: Olaparib reduces cell proliferation in both control and ATM deficient A549 cells. A549-control, A549-CRISPR-ATM and A549-CRISPR-DNA-PKcs cell lines were treated with 4 μM olaparib for 72 or 120 hours then analyzed by the trypan blue exclusion assay. The figure represents the SEM of 3 separate experiments. Statistical significance was determine using one-way ANOVA. ** represents, p <0.001 and *** represents p <0.0001. # indicates statistical significance between A549-CRISPR-control (DMSO) and A549-CRISPR-DNA-PKcs (DMSO), p value< 0.05.

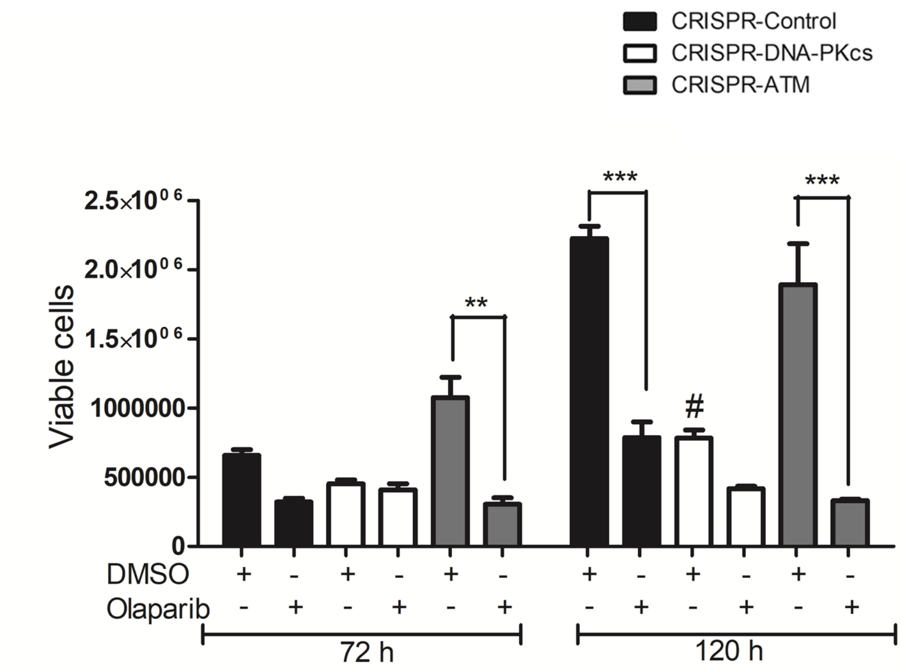

Supplementary Figure 6. Western blots of two additional experiments described and quantitated in Figure 3. A549-CRISPR-control, A549-CRISPR-DNA-PKcs and A549-CRISPR-ATM cells were grown in the presence of 4 μM olaparib for 1, 2 or 4 days as indicated. Samples in lanes 1, 5 and 9 were incubated in an equal volume of DMSO for 4 days. NETN extracts were generated at 50 μg run on SDS PAGE and blotted for the antibodies shown on the right-hand side. Positions of molecular weight markers are shown on the left. Panels A and B represent 2 replicate experiments, the third is shown in Figure 3. The sample in Panel A, lane 13 was control A549 cells irradiated with 2 Gy IR and harvested after 1 hour. The sample in Panel B, lane 13, was A549-CRISPR-control cells treated with nocodazole (40 ng/ml) for 16 hours. Asterisks indicate non-specific bands. See legend for Figure 3 for additional details and Figure 3B-G for quantitation. Panel C shows quantitation for Chk2 T68 phosphorylation. Please see next page.

Supplementary Figure 6

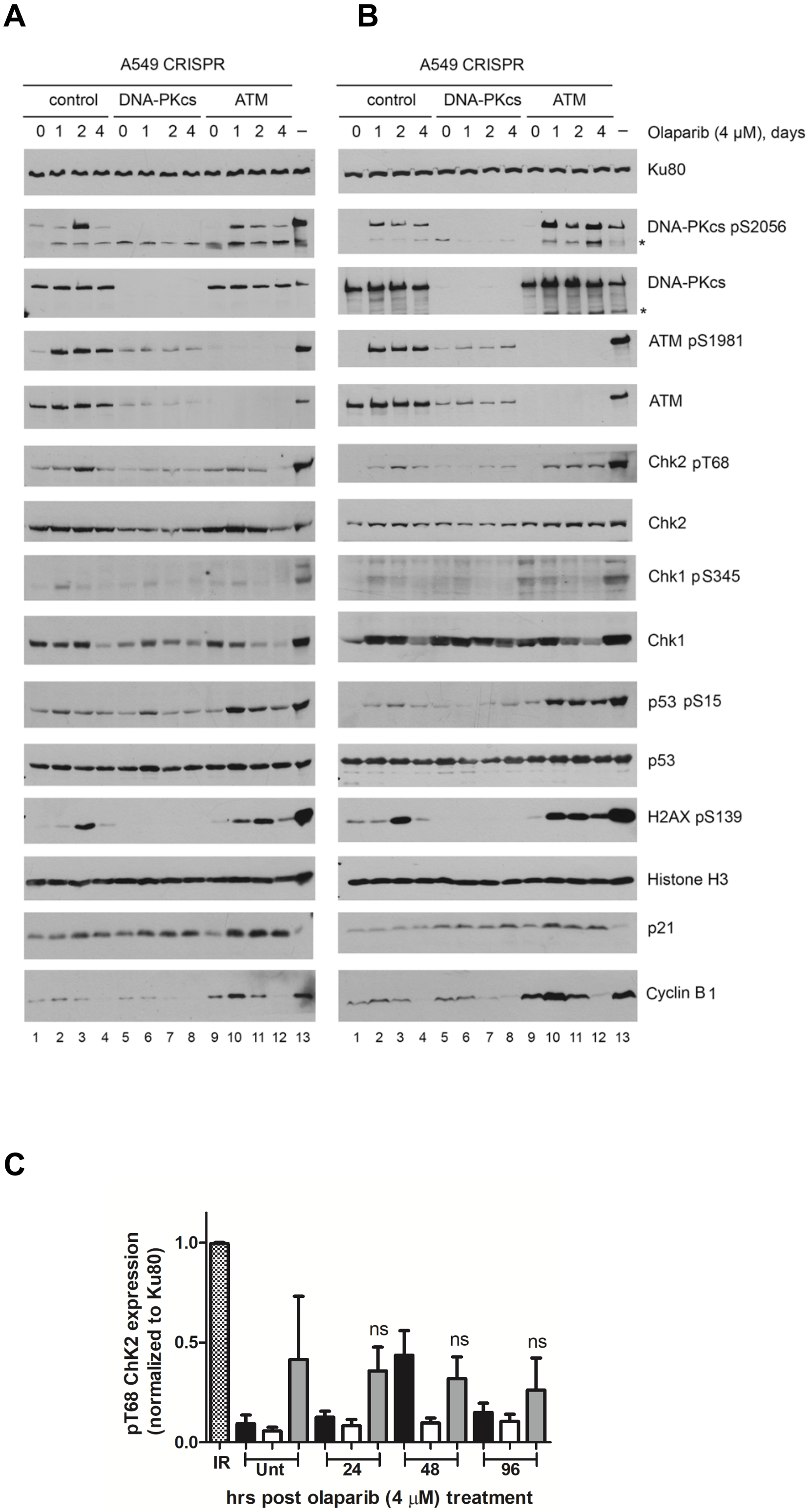

Supplementary Figure 7: Analysis of γ-H2AX foci in olaparib-treated A549-CRISPR-control, A549-CRISPR-DNA-PKcs and A549-CRISPR-ATM cells. A549-CRISPR-control, A549-CRISPR-DNA-PKcs and A549-CRISPR-ATM cells were seeded on poly-lysine coated coverslips and after 24 hours, treated with 4 μM olaparib or an equivalent volume of DMSO and harvested after 1, 2 or 4 days as indicated. Cells were fixed and stained and the average γ-H2AX fluorescence per cell was calculated as described in Supplementary Methods. See **Figure 3** and **Supplementary Figure 6** for corresponding western blots. Statistical analysis was carried out as in Supplementary Figure 4. Please see next page.

Supplementary Figure 7

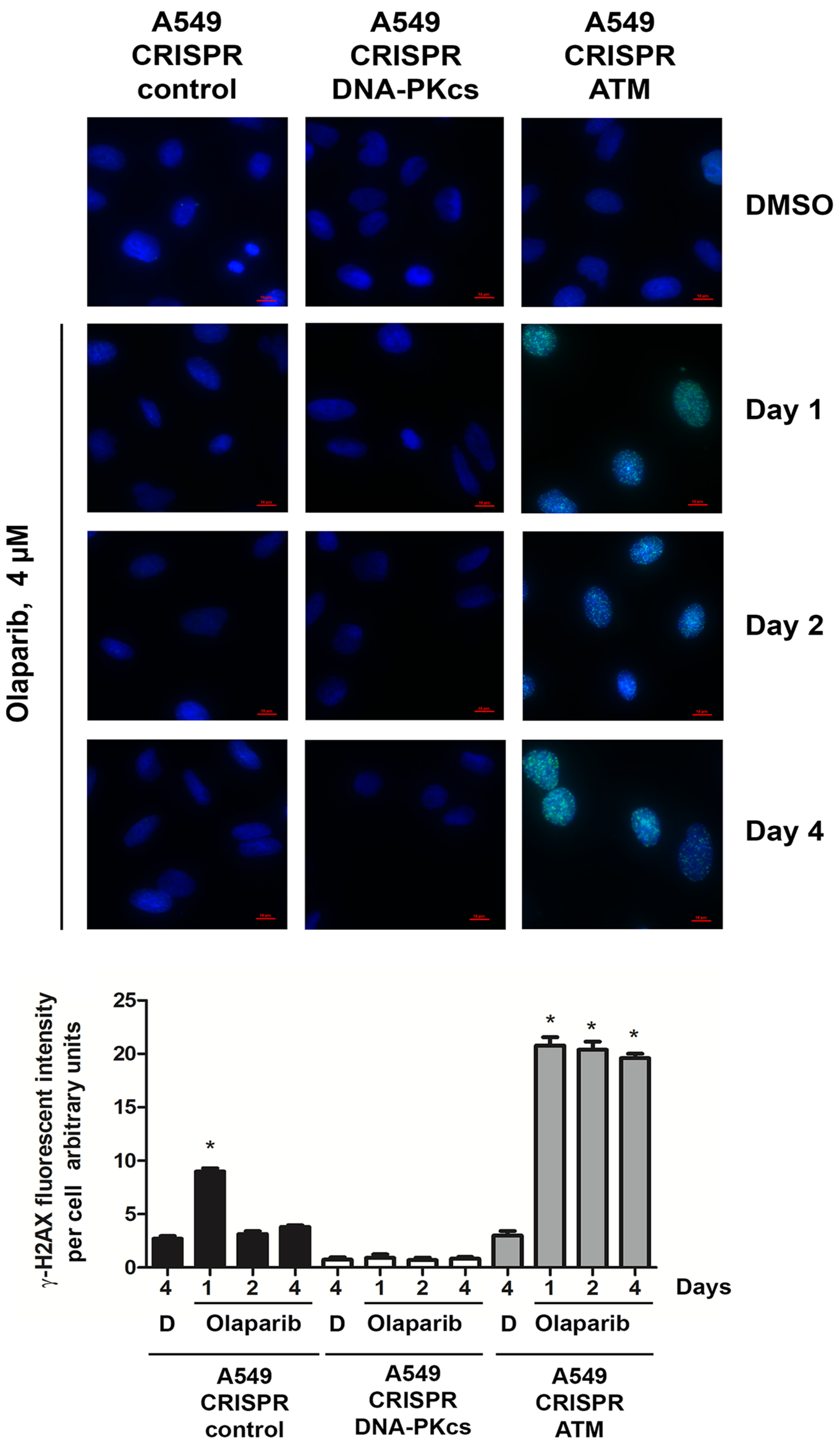

Supplementary Figure 8: Two additional repeats of Figure 4 showing effect of NU7441 and VE-821 on p53, cyclin B and H2AX in A549-CRISPR-ATM cells.

See Figure 4 for details.

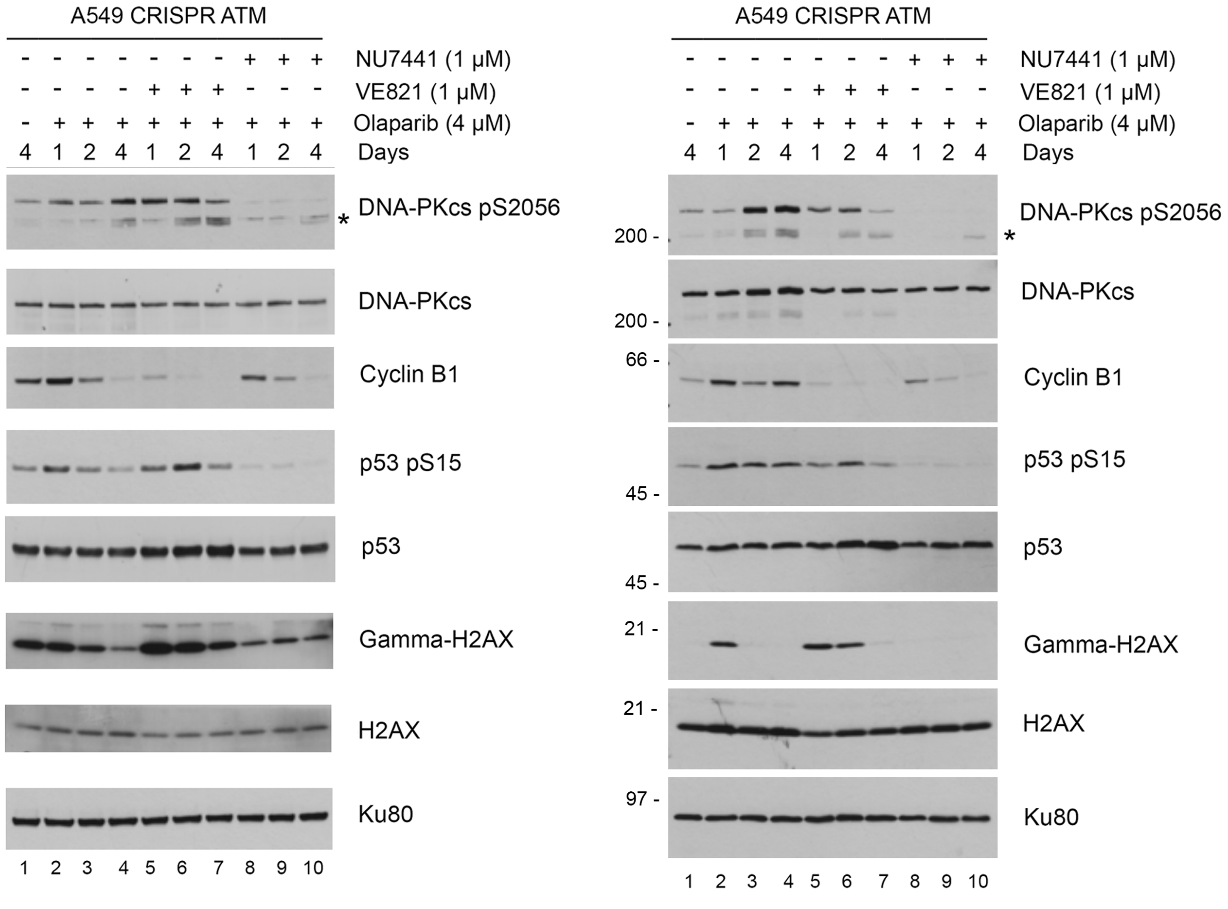

Supplementary Figure 9:

ATM-deficient A549 cells do not undergo apoptosis when treated with olaparib. Cells were treated exactly as in Figure 5A, then analyzed by flow cytometry with annexin staining to determine the percentage of apoptotic events. Staurosporine (1 μM) was used as a positive control. The figure represents the SEM of 3 separate experiments and statistical significance was determined using one-way ANOVA.

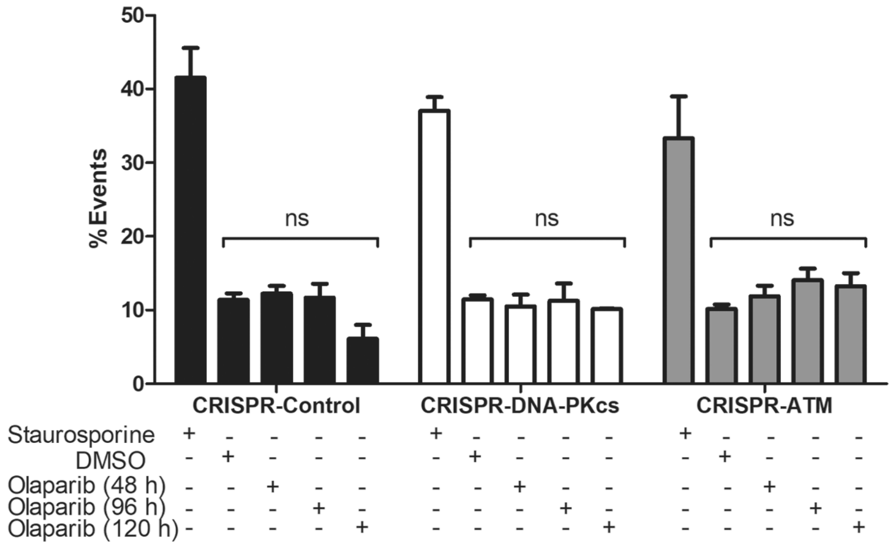

Supplementary Figure 10: VE-821 does not enhance sensitivity of ATM-deficient A549 cells to ionizing radiation.

(A) A549-CRISPR-control and (B) A549-CRISPR-ATM A549 cells were treated with DMSO, 2 μM VE-821 and/or 1, 2 and 4 Gy IR for 48-144 hours as indicated and analyzed using trypan blue viability assays. Results represent the average of three independent experiments. Error bars represent SEM. Statistical significance was determined by one-way ANOVA. None of the combination treatments (IR + VE-821) were found to be statistically significant when compared to IR alone. (C) A549-CRISPR-control and (D) A549-CRISPR-ATM cells were treated with DMSO, 10 μM staurosporine (positive control), 2 μM VE-821 and/or 1,2 and 4 Gy IR for 48-144 hours as indicated and analyzed for sub-G1 population, indicative of cells undergoing apoptosis. Results represent the average of three independent experiments. Error bars represent SEM. Statistical significance was determined by one-way ANOVA. None of the combination treatments (IR + VE-821) were found to be statistically significant when compared to IR alone.

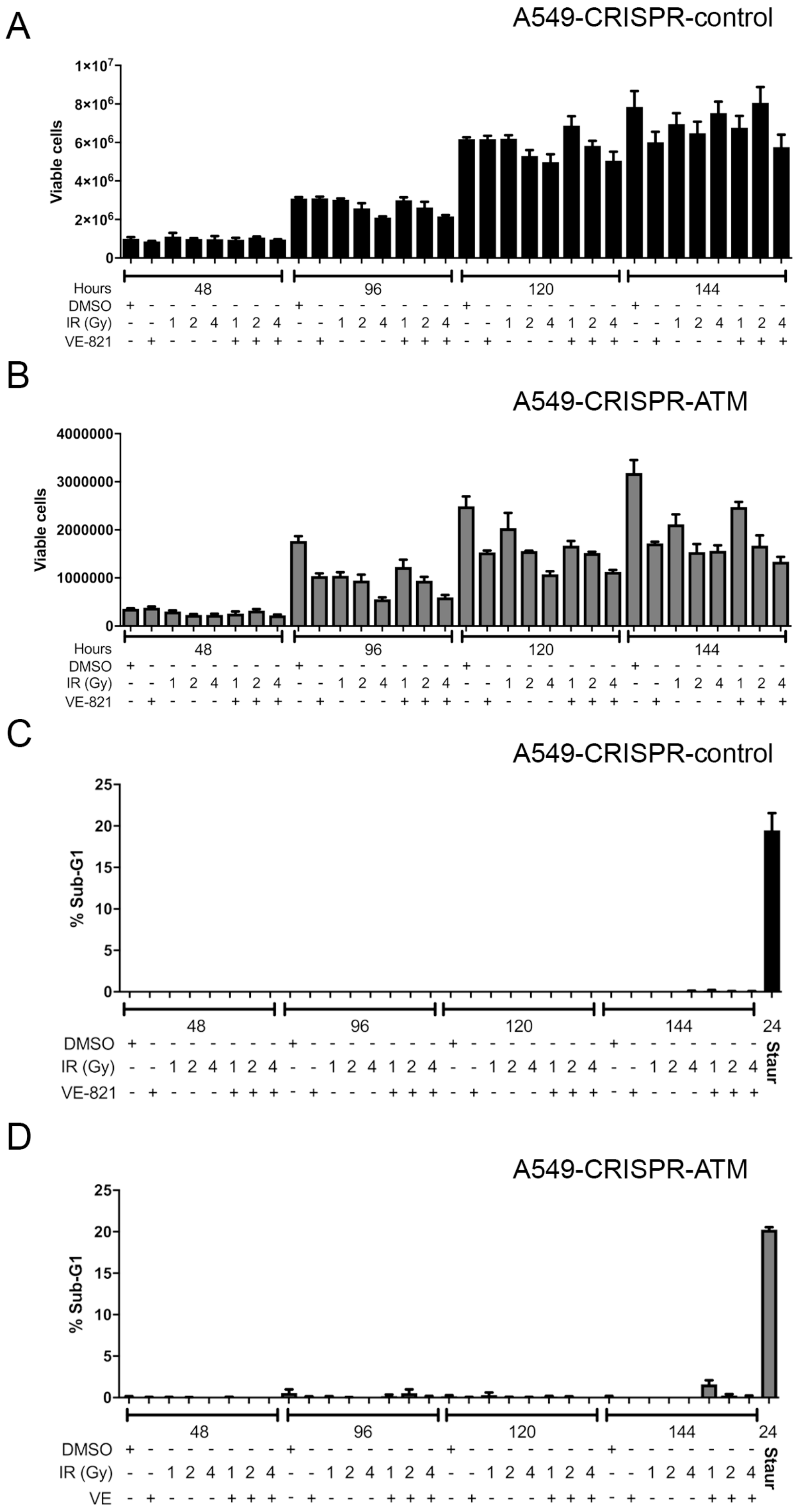
A
A549-CRISPR-control
Viable cells
1×10^7
8×10^6
6×10^6
4×10^6
2×10^6
0
Hours 48 96 120 144
DMSO + - - - - - - - + - - - - - - - + - - - - - - - + - - - - - - -
IR (Gy) - - 1 2 4 1 2 4 - - 1 2 4 1 2 4 - - 1 2 4 1 2 4 - - 1 2 4 1 2 4
VE-821 - + - - - + + + - + - - - + + + - + - - - + + + - + - - - + + +
B
A549-CRISPR-ATM
Viable cells
4000000
3000000
2000000
1000000
0
Hours 48 96 120 144
DMSO + - - - - - - - + - - - - - - - + - - - - - - - + - - - - - - -
IR (Gy) - - 1 2 4 1 2 4 - - 1 2 4 1 2 4 - - 1 2 4 1 2 4 - - 1 2 4 1 2 4
VE-821 - + - - - + + + - + - - - + + + - + - - - + + + - + - - - + + +
C
A549-CRISPR-control
% Sub-G1
25
20
15
10
5
0
48 96 120 144 24
DMSO + - - - - - - - + - - - - - - - + - - - - - - - + - - - - - - -
IR (Gy) - - 1 2 4 1 2 4 - - 1 2 4 1 2 4 - - 1 2 4 1 2 4 - - 1 2 4 1 2 4
VE-821 - + - - - + + + - + - - - + + + - + - - - + + + - + - - - + + +
Staur
D
A549-CRISPR-ATM
% Sub-G1
25
20
15
10
5
0
48 96 120 144 24
DMSO + - - - - - - - + - - - - - - - + - - - - - - - + - - - - - - -
IR (Gy) - - 1 2 4 1 2 4 - - 1 2 4 1 2 4 - - 1 2 4 1 2 4 - - 1 2 4 1 2 4
VE - + - - - + + + - + - - - + + + - + - - - + + + - + - - - + + +
Staur

Supplementary Figure 11: Neither loss of ATM nor olaparib induces reactive oxygen species in A549 lung adenocarcinoma cells.

A549-control, A549-CRISPR-DNA-PKcs and A549-CRISPR-ATM cells were treated with 4 μM olaparib or an equivalent volume of DMSO for 120 hours. Samples were analyzed by flow cytometry with H2DCFDA assay to determine the levels of ROS. Hydrogen peroxide (H_2O_2) (500 μM) was used as a positive control. The figure represents the SEM of 3 separate experiments and statistical significance was determined using one-way ANOVA. The * indicates statistical significance ($p < 0.05$) to other treatment groups within the same cell lines.

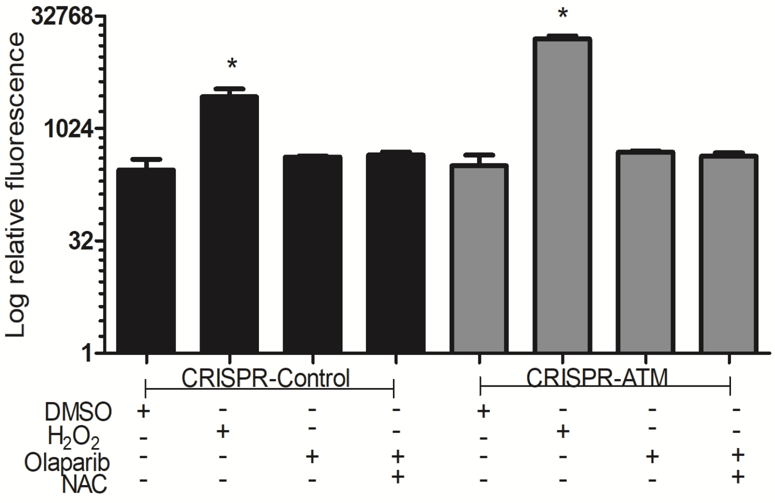

Supplementary Figure 12: Model showing reversible G2 arrest in A549-CRISPR-ATM cells and cell death with the combination of olaparib plus VE-821.

(A) Olaparib and/or olaparib plus VE-821 induce DNA damage in ATM proficient cells but do not induce significant G2 arrest or cell death. (B) In ATM-deficient cells, olaparib induces DNA-PK-dependent phosphorylation of p53 and H2AX and reversible G2 arrest, while addition of the ATR inhibitor VE-821 results in cell death.

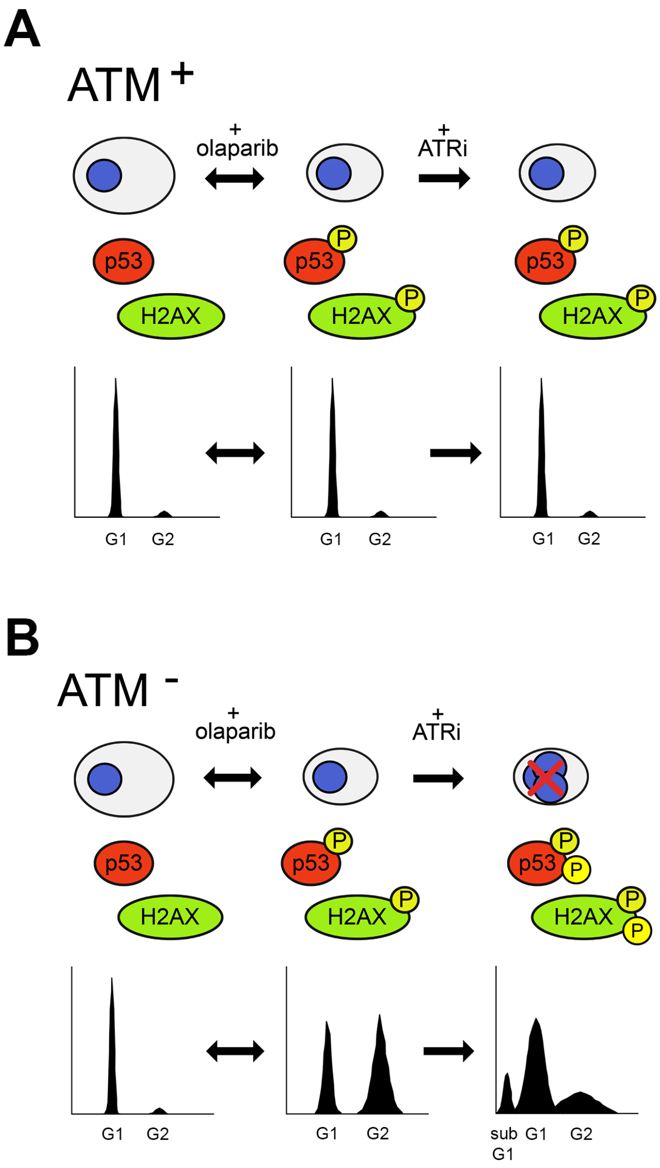

Supplementary references

1. Douglas P, Zhong J, Ye R, Moorhead GB, Xu X, Lees-Miller SP. Protein phosphatase 6 interacts with the DNA-dependent protein kinase catalytic subunit and dephosphorylates gamma-H2AX. *Molecular and cellular biology* 2010; **30**(6): 1368-1381; e-pub ahead of print 2010/01/13; doi 10.1128/mcb.00741-09.
2. Wang C, Jette N, Moussienko D, Bebb DG, Lees-Miller SP. ATM-Deficient Colorectal Cancer Cells Are Sensitive to the PARP Inhibitor Olaparib. *Transl Oncol* 2017; **10**(2): 190-196; e-pub ahead of print 2017/02/10; doi 10.1016/j.tranon.2017.01.007.

Supplementary acknowledgements:

We thank Drs. Lucy Swift and Rima-Marie Wazen (Live Cell Imaging Facility, Snyder Institute for Chronic Disease, Cumming School of Medicine, University of Calgary), for assistance with microscopy and analysis of γ–H2AX foci.

The Nikon Ti Eclipse fluorescence microscope was purchased with funds from the International Microbiome Centre, which is supported by the Cumming School of Medicine at University of Calgary, Western Economic Diversification (WED) and Alberta Economic Development and Trade (AEDT), Canada.
